# Managing Procrastination on Social Networking Sites: The D-Crastinate Method

**DOI:** 10.3390/healthcare8040577

**Published:** 2020-12-18

**Authors:** Abdulaziz Alblwi, Dena Al-Thani, John McAlaney, Raian Ali

**Affiliations:** 1 Faculty of Science and Technology, Bournemouth University, Poole BH12 5BB, UK; jmcalaney@bournemouth.ac.uk; 2 College of Science and Engineering, Hamad Bin Khalifa University, Doha, Qatar; dalthani@hbku.edu.qa (D.A.-T.); raali2@hbku.edu.qa (R.A.)

**Keywords:** digital wellbeing, procrastination, healthy online interaction

## Abstract

Procrastination refers to the voluntary avoidance or postponement of action that needs to be taken, that results in negative consequences such as low academic performance, anxiety, and low self-esteem. Previous work has demonstrated the role of social networking site (SNS) design in users’ procrastination and revealed several types of procrastination on SNS. In this work, we propose a method to combat procrastination on SNS (D-Crastinate). We present the theories and approaches that informed the design of D-Crastinate method and its stages. The method is meant to help users to identify the type of procrastination they experience and the SNS features that contribute to that procrastination. Then, based on the results of this phase, a set of customised countermeasures are suggested for each user with guidelines on how to apply them. To evaluate our D-Crastinate method, we utilised a mixed-method approach that included a focus group, diary study and survey. We evaluate the method in terms of its clarity, coverage, efficiency, acceptance and whether it helps to increase users’ consciousness and management of their own procrastination. The evaluation study involved participants who self-declared that they frequently procrastinate on SNS. The results showed a positive impact of D-Crastinate in increasing participants’ awareness and control over their procrastination and, hence, enhancing their digital wellbeing.

## 1. Introduction

The phenomenon of procrastination is widespread in academia and work contexts, and it refers to the voluntary delay of activities that may prevent people from performing specific tasks, potentially leading to negative consequences [1]. Procrastination has also been defined as the delay of relevant and timely activity [2]. The delay may, therefore, lead to people experiencing negative consequences such as low academic performance, increased levels of stress and anxiety [1,3]. Moreover, procrastination can be considered one of the main sources of work-related stress [4]; for example, it may increase people’s stress due to not meeting deadlines and delivering low-quality work. Several factors may encourage people to procrastinate, such as performing a difficult or mundane task, lack of motivation or lack of energy.

Furthermore, recent technological changes which enable almost unlimited access to the Internet may have played a role in increasing the likelihood of engaging in procrastination, especially for those who use the Internet for their work [5,6]. For example, people can be distracted by notifications that they receive while working, encouraging checking and then procrastination, especially for those who have low self-control. Therefore, the distracting notification can be considered an external factor that could invoke and facilitate procrastination. Moreover, the design features of social networking sites (SNS) could facilitate procrastination in numerous cases such as users who instantly reply to messages to develop a positive self-image or because of the fear of missing out [7,8]. It has been suggested that procrastination is a trait that is determined by underlying personality factors [9]. As such, although SNS may trigger a degree of procrastination in all users, it is possible that some individuals are more likely to procrastinate when encountering these SNS related distractions because they have a greater disposition towards doing so.

SNS platforms enable users to create personal profiles, communicate, and get in touch with others, regardless of their location, experiences, or language. Despite these benefits, such activities tend to become a harmful behaviour for those who engage in excessive online usage, procrastination and digital addiction [10]. Conflict could be argued to be one of the defining characteristics of procrastination in the context of SNS, as it means delaying one task in favour of staying on social media and doing something else [11]. This may be interconnected with digital addiction, where the desire to achieve mood modification leads individuals to use social media as a way to avoid stressful or demanding tasks [8]. Procrastination means a delay of another task and our previous research in [8,12,13] showed that users wish to see motivational elements in that task that then add a degree of satisfaction that they get from social media, for example, being rewarded, task completion and getting social support while doing it.

The possibility of procrastination and delayed work may have increased due to the high level of enjoyment that SNS provide to their users, or due to the pressure that users may feel to respond instantly to meet their contacts’ expectations [8]. SNS features can exacerbate procrastination by providing an immersive and alternative space where users can create and potentially live different personas and roles. While social media has brought many benefits, such usage styles can be seen as problematic, resulting in reduced academic performance, lack of real-world social skills, neglecting meals and physical activities. Our previous work has also demonstrated that the design of SNS features plays a significant role in facilitating procrastination [8,12,13]. This indicates that a method to combat procrastination on SNS needs to build resilience to these SNS triggers and utilise additional features to combat them. Hence, solutions to procrastination shall be two-fold. First, a resilience towards social media temptation needs to be in place and, second, the reason for the delay and how to reinforce performance shall be a factor in the design and success for any tool to combat procrastination.

In this paper, we propose the D-Crastinate method to aid users to be conscious and in control of their procrastination on SNS. In our previous work, we demonstrated the role of SNS design in facilitating procrastination and explained the different types of procrastination [8]. We suggested countermeasures to combat procrastination and how to implement these countermeasures in SNS design [12]. We then conducted a survey involving 334 participants to examine the extent to which the respondents agree with our previous findings [13]. Based on the results of the previous studies and other theoretical underpinnings (Section 2), we developed the D-Crastinate method to combat procrastination on SNS (Section 3). The D-Crastinate method is intended to: (i) raise users’ awareness of procrastination on SNS; (ii) guide users to identify the features that facilitate their procrastination as well as their procrastination types; (iii) suggest personalised countermeasures based on the triggering SNS features. We assess the D-Crastinate method against four aspects: clarity, coverage, procrastination awareness, acceptance, and potential (Section 4).

## 2. Theoretical Underpinnings

In this section, we discuss the theories that informed the design of D-Crastinate. These theories and concepts are social norms, poor expectation management, impulse control, time management, habitual checking, the health belief model, the transtheoretical model, relapse prevention, digital resilience, positive thinking, managing relatedness and acceptance of imperfection.

### 2.1. Social Norms

Social norms refer to what we perceive to be the typical behaviours and attitudes of our peers. The literature of social norms considers two types of social norms, descriptive norms, and injunctive norms. Descriptive norms refer to the observable behaviour, whereas injunctive norms refer the attitude held toward a particular behaviour [14,15]. For example, a perceived descriptive norm amongst students may be that most of their peers check SNS whilst in lectures, with the perceived injunctive norms being that most students think this acceptable. It is important to note however that what someone perceives to be the norm may not be the actual norm. Social norms research has demonstrated that individuals believe that others tend to behave in a more negative way than they do themselves, and that others hold more negative attitudes [16]. For example, it has been found that individuals tend to assume that others drink more alcohol than is actually the case, and also that they overestimate how accepting their peers are of heavy alcohol consumption [15,17].

Given that people tend to assume that others behave more negatively than they do themselves, it is possible that procrastination in others is also overestimated. This type of thinking might prevent users from seeking solutions to control their procrastination. Applying social norms approach on perceived descriptive or injunctive norms can promote healthier behaviour and encourage help-seeking where users can realise the issue of procrastination on SNS [18,19].

### 2.2. Poor Expectation Management

The findings of our studies have demonstrated that procrastination on SNS could occur due to peer pressure where users might believe they have to meet their peers’ expectations to maintain popularity and to build a positive image [8,13]. Therefore, managing others’ expectations can play a significant role to reduce that pressure, eventually reducing the possibility of procrastination. Setting others’ expectations can be achieved by declaring some information regarding user’s availability, or the tasks that users are currently performing, and making those situations transparent to peers. Confirming the availability time can also reduce the fear of missing out (FoMO) where users fear being ignored or excluded [7,20]. Being transparent can inspire and create trust between users and provide an excuse to avoid interacting with others during the unavailability time. On the other hand, transparency of users’ availability could also introduce privacy risks [21,22]. However, the risks can be mitigated by the way transparency is operated, and SNS typically allow restriction on who can see information like status and posts.

### 2.3. Impulse Control

Impulsivity refers to the unplanned reaction to external or internal stimuli without considering the negative result of these reactions to the individual or others [23]. People who are keenly oriented towards the present result of the actions may not consider the long-term consequences and how it could be harmful to them. Barratt and their colleagues have developed the most widely used model of impulsivity behaviour [24,25]. The model considered impulsivity as a unidimensional factor that is orthogonal to anxiety [25].

Procrastination is associated with impulsivity behaviour [3,26]. In impulsive behaviour, people might act without thinking, and they might take risks for seeking immediate pleasure. Numerous studies have demonstrated the importance of considering emotion as a trigger of procrastination, such as the work in [27,28]. The immersive design of SNS, where the content and interfaces are personalised based on the users’ interest and preference, has a significant impact on increasing the procrastination time and fulfil the user gratification [29,30]. There is a need to educate users on how to increase control of their impulse behaviour. Various strategies can be used to increase the user’s awareness about impulsivity and to help them to think before acting, e.g., through teaching mindfulness strategies. Some countermeasures in D-Crastinate are meant to increase the user’s awareness of their level of impulse control. They provide feedback about procrastination time and suggestions to help to manage or rethink it. Technical countermeasures such as a universal block of social media apps or their notifications when one wants to focus are additional help to control the impulsivity.

### 2.4. Poor Time Management

Time management refers to the process of determining needs, setting goals to accomplish these needs, and prioritising the tasks required to accomplish these goals [31]. Time management also refers to the technique for effective time use [32], or the planning and allocating time for each task to be achieved [33]. Procrastination can be reduced by applying time management tools, and these tools can also have a positive impact on reducing the stress associated with procrastination [34]. However, some SNS features have temporarily available content which can also be seen as a trigger of procrastination as users must frequently check it, otherwise it either disappears or becomes outdated. SNS suggest more content when a user checks with a particular goal in mind, leading to spending extra time on it. Our suggested countermeasures pay particular attention to helping users to manage their time better. We suggested different tools such as usage feedback which provides specific information about procrastination such as the time spent and the apps that were most used [12,13]. This could help users to have a greater awareness and control of their usage. These countermeasures help to guide users on how to set their goals and the time they wish to spend on SNS. Our countermeasures also considered the side effects of resisting temptation such as stress resulting from receiving multiple reminders in a short time. Users are encouraged to decide the time that they wish to receive reminders and the quantities of those reminders. There are existing tools in the market aimed at helping such self-monitoring and limit settings. Google Digital Wellbeing and iOS Screen Time are examples.

### 2.5. Habitual Checking

Habitual checking refers to the automaticity access and use of SNS due to the gratification that users received from such actions [35]. Over time, the behaviour becomes action-scripts that users perform without conscious reflection on the reason for doing the action and its consequences [35,36]. Some researchers conceptualised the habit as a type of gratification [37,38]. However, the gratification seeking on SNS is a predictor for the compulsive and excessive usage which over time leads users to a habitual checking where the use of SNS becomes uncontrolled [39,40]. Diversion and relationships building are factors for gratification that can leads to the habitual checking, where users access SNS to seek pleasure and entertainment [41]. Self-presentation is also seen as a trigger for gratification [42]. Self-presentation refers to information management in which users chose how to represent themselves to others. SNS features play an essential role in increasing gratification seeking, including the identity, and profiling features and the immersive design. These features increase the urge to seek gratification that could lead users to access SNS unconsciously and to a habitual checking.

### 2.6. Health Belief Model

The health belief model (HBM) focuses on the relationship between beliefs and health. HBM suggested that preventive health behaviour consists of personal beliefs [43]. There are six components for HBM, which include perceived severity, perceived barriers, perceived susceptibility, self-efficacy, perceived benefits, and cues of action [43]. The way that people relate themselves to these components is predictive of whether they are engaged in particular actions or behaviours. However, the D-Crastinate materials which will be provided to users can increase their awareness which can change the way users think about procrastination. These materials also illustrate how users can overcome the barriers and ensure that users get prepared for the procrastination. These materials consider the HBM to change users’ beliefs about procrastination which can positively affect the behaviour of the users toward the procrastination on SNS. For example, peer pressure is one of the barriers that may lead users to procrastinate to meet their peers’ expectations. We can address that barrier by using the show availability countermeasures, which help the user to manage their peers’ expectations regarding the time in which peers can expect the replies. This can reduce the pressure to respond immediately that the user may feel.

### 2.7. Relapse Prevention

Relapse refers to the failure in individuals’ attempts to change or moderate a targeted behaviour. Relapse prevention refers to the strategy to ensure that the person will keep greater control over their change process and not be back to the addictive behaviour again [44]. Relapse can occur in multiple stages, including emotional relapse, mental relapse, and physical relapse [45]. Emotion relapse can occur on the earlier stage where individuals start to think about the addictive behaviour and how it could help them to cope with their situation such as stress and need to change the mood, i.e., smoking. The second stage of the relapse is the mental stage where there is conflict inside the individual. Finally, physical relapse happens when the person is back to addictive behaviour [45].

It is hard to prevent the relapse of procrastination on SNS because of the availability and the ease of the accessibility to smartphones most of the time. Personalised content which is based on user interest and the temporary content such as Snapchat stories are features that increase the possibility of the relapse to procrastination. Moreover, a relapse might happen when users procrastinate to change their mood and/or cope with stress associated with a task. The instantaneous gratification that SNS design provides such as likes or positive comments trigger relapse and could significantly affect control over the procrastination. However, our suggested countermeasures are meant to consider these design triggers and decrease the possibility of having a relapse. In D-Crastinate, we also took a further step to educate users about how relapse occurs and provide them with guidelines for relapse prevention. The guidelines focus on motivating users to finish the process and ensure that users would not deviate from their goals. Users are required to identify their goals and motivation for using this method as a prerequisite. Then, reminders and suggestion are offered during the method stages.

Other strategies that could help relapse prevention include peer support, learning from setbacks, and having a positive self-labelling. Using these strategies can increase the user’s self-esteem and self-efficacy to moderate and reduce the possibility of the relapse and eventually have greater control over procrastination time.

### 2.8. Digital Resilience

Resilience, in general, refers to the ability that an individual has to deal effectively with changes and threats, and the ability to recover quickly from challenges and difficulties [46]. Building digital resilience will enable users to have the resources that help them to deal with online issues such as procrastination. These resources could include understanding when the user is at risk, information on how to seek help, and guidelines for recovering quickly from issues’ side effects. To increase digital resilience, we could offer users timely feedback to help them to understand their current situation about procrastination. This feedback can also provide more suggestions for users on how to control procrastination. High digital resilience entails advanced defence skills where people can combat procrastination triggers and eventually control procrastination better. Digital resilience can be built for users by illustrating how procrastination happens on SNS and the features that facilities it.

### 2.9. Positive Thinking

Using a positive thinking strategy is suggested to increase users’ self-efficacy, which would increase their confidence in the ability to resist social media triggers and combat procrastination [47]. Our suggested countermeasures provide users with information focussing on their improvement in controlling their procrastination and relapse prevention. Over time, this is hoped to enhance users’ self-concept and keep users motivated. Additionally, the framing of the content when suggesting countermeasures is expected to play an important role in increasing motivation and positive thinking.

### 2.10. Relatedness and Connected to Others

Relatedness is a component of the self-determination theory, and one of the three needs that people should have to be motivated [48]. One of the reasons for which people may delay their current tasks is to meet the expectations of others and be responsive to their communications. As demonstrated in our previous work in [12], SNS offer tools to increase social interaction, but while doing so, they may increase the pressure on users to respond and be excessively online. This triggers FoMO, i.e., the desire to check and be aware of what is happening [7], as well as procrastination, i.e., to prolong the time spent online despite having to be doing something else. Hence, by adding novel techniques for managing expectations of social media design, users can still relate to others without feeling pressure to do so, and can interact in a more managed style. Examples of such techniques include a chat timer, advanced versions of auto-reply and a chat progress bar [12].

### 2.11. Acceptance of Imperfection

Procrastination has a strong relationship with perfectionism where people are online excessively due to the desire to enhance their online persona and reply to all requests on SNS instantly to maintain a positive self-image [49]. In [8], the authors suggested that people might procrastinate on a task by replying to messages instantly to maintain a positive relationship with others and to build a positive self-image. The perfectionism model suggested by [50] included six dimensions; personal standards, parental expectations, parental criticism, concern about mistakes, doubts about actions, and organisation. When people try to satisfy these dimensions on an SNS, it can lead them to neglect other priorities and make other tasks hard to achieve. However, the acceptance of non-perfectionism can reduce the feeling of being criticised or evaluated by online peers and positively lead users to learn from previous mistakes without pressuring themselves [51].

### 2.12. Transtheoretical Model

The transtheoretical model, also known as the stages of change model, states that behaviour change occurs through a series of steps [52]. These are precontemplation, contemplation, preparation, action, and maintenance. This model has been used extensively within psychotherapy, particularly in relation to addictive behaviours [53]. Part of the basis of the model is that individuals must first recognise that they have a problematic behaviour and have the motivation to bring about a change in this behaviour. There have been criticisms made of how well the model applies to behaviour in real-world settings; nevertheless it remains an influential conceptualisation of how behaviour change should be approached [53].

## 3. D-Crastinate Method Stages and Its Guidance to Use

We developed the D-Crastinate method stages based on the results of the exploration, co-design, and confirmation phases [8,12,13], and also as informed by the research literature on behaviour change. The D-Crastinate method targets people who self-declare that they frequently procrastinate on SNS and are willing to utilise such countermeasures to reduce their procrastination. This is consistent with the transtheoretical model of behaviour change, in which individuals must first recognise that a behaviour is problematic and wish to change this before action can be taken. D-Crastinate relied on self-declaration given the lack of standardised measures for procrastination. Table 1 presents the stages of D-Crastinate and their expected outcomes. D-Crastinate stages include education, self-diagnosis, planning and preparation, action, self-assessment, and error identification. In Table 2, we guide users on how to apply the D-Crastinate method. In the following sub-sections, we elaborated each stage of the D-Crastinate method. The user-friendly version of the method can be found in the Booklet provided as a Supplementary Materials of this paper. In the Booklet (Page 12), users can also find some of the suggested software tools that can help to monitor procrastination time or even in controlling procrastination through setting limits on screen time as a whole, the time they spend on certain applications and the number of times they unlock the smartphone.

### 3.1. First Stage: Education

In this stage, D-Crastinate educates users about the phenomena of procrastination, associated consequences, and the negative results of it (see page 1 to 8 in the Booklet). This stage is to increase users’ awareness of procrastination and build digital resilience to it. D-Crastinate provides guidelines on how to use the D-Crastinate method and information about each stage (see Page 2 and 3 in the Booklet). By the end of this stage, users should learn the main concepts of countermeasures, procrastination types, and the SNS features that could trigger procrastination. Users will also be provided with an explanation about relapse and how it can prevent them from completing the process of the interventions. The relapse can occur in any stage and at any time. Therefore, users must prepare for it to complete the use of the proposed method successfully. D-Crastinate helps relapse prevention by motivating users to complete the use of suggested countermeasures and reduce procrastination time. In the education stage, the method provides participants with additional countermeasures providing alternative strategies to combat procrastination on SNS. In the booklet, we used a language that is easy to grasp.

### 3.2. Second Stage: Self-Diagnosis

The first self-diagnosis task concerns the identification of the types of procrastination faced by the user. The second task concerns the identification of the SNS features that facilitate their procrastination.

#### 3.2.1. Self-Diagnosis for Procrastination Types

In this task, D-Crastinate helps users to identify their procrastination types (see Table 3) and (see Page 9 in the Booklet). This part can help to understand the user’s motivation for procrastination which includes avoidance, emergence, mood modification, and escapism [8]. This identification of the types of procrastination shall play a significant role in increasing users’ awareness and building their digital resilience.

#### 3.2.2. Self-Diagnosis for SNS Features that Facilitate Procrastination

In this task, users will need to identify the features of SNS that are perceived to facilitate their procrastination (see Page 10 in the Booklet). Table 4 identifies families of SNS features that trigger procrastination. These features play a significant role in increasing the likelihood of procrastination [8]. Once the users successfully identified the features of SNS that trigger their procrastination, they can move to the next stage of the planning and preparation. We note here that these countermeasures exist in various forms in the different SNS and external software or plug-ins to manage online time. We refer to them at the category level rather than referring to specific tools available in the de-facto social media and commercial tools.

### 3.3. Third Stage: Planning and Preparation

This stage has two phases which include tasks engagement tools and procrastination countermeasures. Firstly, users can select tools to motivate them to keep focusing on their tasks (see Table 5) [12]. These tools are to bring the joy that users might have in SNS to the delayed tasks (see Page 11 in the Booklet). Additionally, D-Crastinate provides users with customised and personalised countermeasures to combat procrastination (see Table 4) [12]. The customisation process will be based on the selected features that trigger procrastination which has already been identified in the previous stage. In the D-Crastinate booklet, and as a visual aid, the same background colour was used to match the SNS features that lead to procrastination with their customised countermeasures (see Pages 12 to 14 in the booklet). Users can select different countermeasures if they wish. Once the users identified the preferred countermeasures to use and the tasks motivation tools, they can move to the action stage.

### 3.4. Fourth Stage: Action

In this stage, D-Crastinate requires users to apply the suggested task engagement tools and procrastination countermeasures for some time, typically one week (see Page 15 in the Booklet). As the process is iterative, D-Crastinate suggested one week for the first iteration to test the acceptance of the countermeasures and evaluate whether they helped users to improve their control over their procrastination. The users can then either continue the same countermeasures, remove some and elect additional ones. The users will monitor their control over procrastination while using the proposed tools and countermeasures. D-Crastinate provides diary sheets for self-reflection (see Self-monitoring Sheet, Page 23 in the Booklet). As soon as the action stage is finished, the users will move to the self-assessment stage.

### 3.5. Fifth Stage: Self-Assessment

In this stage, D-Crastinate provides a survey to users to self-assess their feedback about the used countermeasures and whether it helps them to gain more control over their procrastination (see Table 6 and Page 16 in the Booklet). If users fail to get a noticeable control over their procrastination or they do not notice any improvement, they are advised to move to the next stage (error identification). The mission of D-Crastinate is completed if users start to gain control and engage with the countermeasures. The mission of the method is to promote a change and serve as a trigger for it. Sustaining the change would require more stages and aiding mechanisms. The long-term counselling and mentoring remain beyond the scope of D-Crastinate. The method advises users to iteratively check and re-assess their procrastination. We note here that D-Crastinate provides users with education about relapse and other literacy materials that are meant to help their long-term effect and digital resilience.

### 3.6. Sixth Stage: Error Identification Stage

In this stage, the users are expected to answer some questions should the previous stages indicate little control over procrastination. These questions are meant to help users to re-identify their types of procrastination and the features that facilitate it (see Page 17 in the Booklet). Once the user answered the following questions, they will be able again to select more specific countermeasures to help control their procrastination.

1. Where did you procrastinate? (In which application)
2. When did you procrastinate? (What time)
3. What did you miss? (Tasks that you missed)
4. Why did you procrastinate? (Your reasons for procrastinating)
5. How did you procrastinate? (Other activities you did while procrastinating)
6. Who did you procrastinate with? (Other people who were involved and who were perhaps affected by your procrastination)

## 4. Evaluation of the D-Crastinate Method

In this section, we evaluate whether D-Crastinate works effectively to improve users’ control over their procrastination. We emphasise that the mission of the method is to trigger a change, while sustaining it would require additional stages and tools.

### 4.1. Evaluation Method

We evaluated D-Crastinate in terms of its clarity, coverage, procrastination awareness, acceptance, and potential to trigger a change. The evaluation study adopted a mixed-methods approach in which we used the qualitative measures of focus group and diary study, together with the quantitative measure of a survey. The combination of qualitative and quantitative data could offer the study more insight, better understanding and the ability to consolidate its outcomes [54,55]. The study involved participants whowere aged over 18 years and who self-declared frequent procrastination on SNS and desire to control it. We conducted the evaluation process in three stages.

In the first stage, we provided our participants with an explanation about procrastination on SNS and the strategies and tools to combat it. The participants were encouraged to share their stories about procrastination and how it affected both their wellbeing and their academic or work performance. Sharing stories about procrastination has also been meant as a warm-up activity. It also helped to ensure that all of the participants were engaged in the session and to provide more in-depth insights into how the design of SNS may facilitate procrastination [56]. At the end of this stage, the participants were asked to apply their own strategies to control procrastination for three days. In other words, they were asked to try and control their procrastination based on explanation of the behaviour and tools existing in the literature to combat it. We did not offer D-Crastinate artefacts in this phase.

In the second stage, the participants discussed both the usefulness and limitations of their chosen strategies and explained whether these strategies had helped them to control their procrastination better. Then, the participants were asked to fill in the self-report questionnaire, which is meant to help them to identify their types of procrastination and the features of SNS that may facilitate this procrastination (see Appendix A). After that, we provided a more detailed presentation that included the types of procrastination; the features that trigger procrastination; the suggested countermeasures to combat procrastination on SNS. Subsequently, we explained the D-Crastinate method and its stages and answered their queries. Finally, the participants were asked to follow the D-Crastinate method and its materials for one week (see D-Crastinate Booklet provided as a Supplementary Materials to this paper).

In the third stage, the participants handed back the materials that were provided to them in the induction session with their choices’ comments included. In a follow-up session, the participants also discussed the usefulness of the D-Crastinate method and whether it had helped them to control their procrastination better than the ad-hoc strategies adopted in the first stage. At the end of the session, the participants filled in the e-TAP scale and they also filled in the second self-reporting questionnaire (see Appendix B and Appendix C). The e-TAP scale was used to measure the participants intention and attitude to use the D-Crastinate in the future [57]. For the full study design and ethics approval as well as samples of the participants’ answers and diaries please refer to Chapters 7 and 8 of [58]. We note that an earlier name of the method was CPoSNS but based on users’ feedback and to communicate the purpose of the it in a more intuitive way, we renamed our method to D-Crastinate.

The collected diaries and the participants’ comments on the questionnaire were transcribed and cleaned up. A content analysis was applied to the qualitative data that were written either in the diary or in the survey. Descriptive and inferential analyses were applied in the quantitative part of the survey. A Shapiro–Wilk test of normality determined that several of the questionnaire items were non-normally distributed. As such, Wilcoxon signed-rank tests were used to determine the effectiveness and usefulness of the proposed method. Wilcoxon signed-rank tests were used also used to determine the statistical significance of the changes after the delivery of D-Crastinate.

### 4.2. Findings

A total of 30 participants took part in the evaluation study: 13 (43%) male and 17 (57%) female. The participants’ ages ranged between 19 and 41 years (mean = 25.63 and standard deviation = 5.58). All of the participants self-declared to procrastinate frequently on SNS and their desire to change this behaviour.

#### 4.2.1. Clarity of D-Crastinate

**Figure 1:**
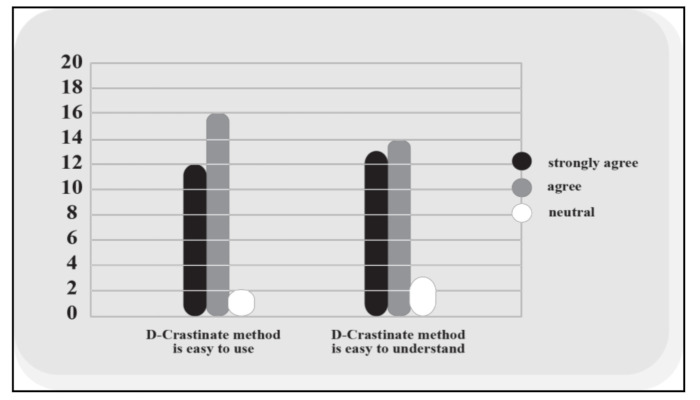
Clarity and ease of D-Crastinate.

Concerning the extent to which the participants found the D-Crastinate method and its materials easy to understand, 27 (90%) of the participants either agreed or strongly agreed with the assumption that the D-Crastinate method was not difficult to understand (see Figure 1). However, three (10%) of the participants selected the neutral option. Some participants reported that some of the colours used in the booklet made the text a little hard to read and they suggested making the text background brighter. An example of these comments is, “Colours could be brighter, especially the purple one”. In general, the comments about the structure were positive and highlighted the benefit of using shapes and colours to match the content. The following are examples of comments about the structure and presentation of the D-Crastinate content:

- “Nicely organised and clear guidance.”
- “Good use of coordinated colours to make it fun.”
- “Good use of diagrams to avoid it being too text-heavy.”
- “It helped me understand how and why we procrastinate. This information was very clear and engaging.”

In response to the question about whether D-Crastinate was easy to use, 28 (93%) of the participants chose either ‘strongly agree’ or ‘agree’. Twelve (40%) of respondents chose ‘strongly agree’; 16 (53%) participants chose ‘agree’; two of the participants chose the neutral option (see Figure 1). The participants reported that the D-Crastinate was easy to apply, and the guidance did not need any further explanation. For example, one participant said, “Very well explained, I didn’t need to question anything throughout the week”. However, another participant reported that the time that was given to apply the D-Crastinate was short as they said, “No difficulties; however, the intervention duration was quite short to able to explore other aspects of it”. Meanwhile, another respondent said, “The countermeasures were very good and easy to use; the use of D-Crastinate just made me a lot more aware of what I could do to combat procrastination”.

#### 4.2.2. Coverage of D-Crastinate

**Figure 2:**
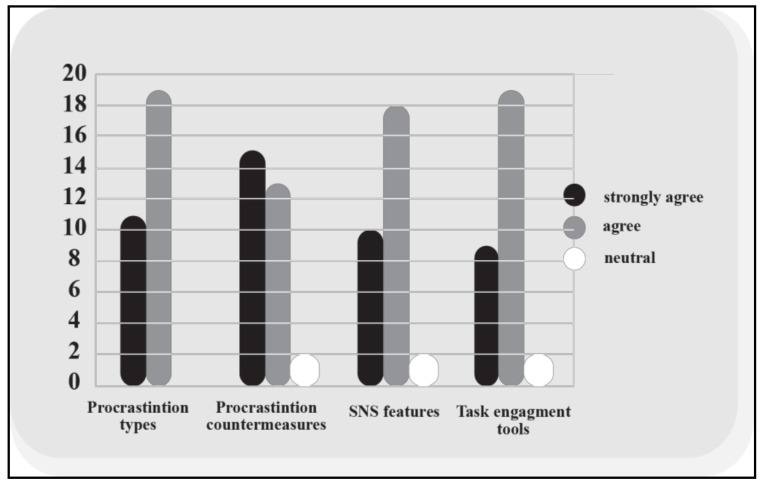
Coverage of D-Crastinate.

D-Crastinate provides information about procrastination and its types, SNS features triggering it, task engagement tools and procrastination countermeasures. In response to the question of whether the D-Crastinate method in general provided sufficient information, all the participants either agreed or strongly agreed with this assumption. Twelve (40%) of the participants strongly agreed, and 18 (60%) of the participants selected ‘agree’ (see Figure 2). Overall, the participants reported that D-Crastinate and its materials had a clear structure and provided sufficient information about SNS procrastination in its various facets and the use of the method. One respondent said, “The method seemed simple and easy to follow”. D-Crastinate simple structure and content helped to increase user engagement and reduced the threat of users failing to complete the entire process of the method. One participant said, “It was good that the booklet even gave instructions for how to set auto-reply and other countermeasures”.

Figure 2 showed that all of the respondents either agreed or strongly agreed that D-Crastionate provided them with sufficient information about the types of procrastination. Eleven (37%) participants selected ‘strongly agree’, while 19 (63%) of the participants chose ‘agree’. Educating the participants about the four different types of procrastination is meant to help users to comprehend the complex concept and allow them to customise measures to combat their particular type. One of the comments was that such education could in “itself be part of the solution to reducing procrastination”.

Figure 2 shows that respondents were generally in agreement on whether they received sufficient information about the SNS features that facilitate procrastination. Most respondents (28; 93%) either strongly agreed or agreed with this statement, while two (7%) selected neutral. Combined with the general agreement on ease of use discussed earlier, this coverage agreement also means that participants could successfully pinpoint the features that usually lead them to procrastinate. One participant said, “It helped me to understand how and why we procrastinate. This information was very clear and engaging”. Another participant said, “Now that I’m more aware of these features, I’m able to notice which ones trigger me to procrastinate more and try to avoid that happening”.

Concerning the task engagement tools, Figure 2 demonstrated that 28 (93%) of the respondents either agreed or strongly agreed that they had received sufficient information about the tools that could increase their motivation to keep focusing on their tasks and minimise the risks of procrastinating on SNS. Meanwhile, two (6%) of the respondents selected the neutral option. Examples of task engagement tools are rewards and reduction, which may provide extrinsic motivation for users and thereby increase commitment to the task.

Concerning the countermeasures proposed to combat procrastination, 28 (93%) either strongly agreed or agreed that D-Crastinate offered sufficient information. A total of 15 (50%) of the respondents chose ‘strongly agree’ and 13 (43%) chose ‘agree’. D-Crastinate pairs the countermeasures with the SNS features that facilitate or encourage procrastination, so each feature has its own countermeasures. We colour coded that pairing as a visual aid. One user said: “Well explained countermeasures, having different shapes and colours made it easy to follow”.

#### 4.2.3. Procrastination Awareness

To evaluate the improvement of the awareness of procrastination on SNS, we compared the participants’ answers before and after applying the D-Crastinate method. The participants self-reported their level of awareness about how procrastination happens on SNS, as well as rating their awareness of how to control procrastination before and after applying the D-Crastinate method. Table 3 demonstrates the change in procrastination awareness. Before using D-Crastinate, 10 (33%) of the respondents selected ‘Yes’, 13 (43%) of respondents selected ‘Not Sure’ and seven (23%) were not aware of how procrastination happens or how the design of SNS could lead them to procrastinate. After using D-Crastinate, 29 (96%) of the respondents became aware of how the design of SNS could trigger their procrastination, and they began to realise how to prevent procrastination from happening.

Specifically, the participants were asked whether they were aware of the features of SNS that facilitate their procrastination. Table 7 shows a comparison of this awareness before and after applying the D-Crastinate method. It can be noted that 29 (96%) became very aware of the features that trigger procrastination on SNS. Concerning how the participants rated their awareness about how to control procrastination on SNS, Table 7 compares the results before and after using D-Crastinate. It can be noted that only six (20%) respondents were moderately aware of how to control procrastination before using D-Crastinate method; however, this number significantly increased to 26 (86%) after using the D-Crastinate.

#### 4.2.4. D-Crastinate Potential

To assess the usefulness of D-Crastinate in triggering a behaviour change, we compared the participants’ procrastination experience before and after using the method (see Table 8). We conducted Wilcoxon sign-ranks test regarding the experience of each type of procrastination and each feature of SNS that leads to it.

Regarding the experience of avoidance type of procrastination, where people procrastinate to avoid doing other work, the result of the Wilcoxon signed-ranks test demonstrated a significant change. Before applying the D-Crastinate, the result was (mdn = 4) and after (mdn = 3) using D-Crastinate: Z = −4.43, *p* < 0.001.

Concerning the experience of mood modification type of procrastination, where people procrastinate to change their mood and to feel better; the result of the Wilcoxon signed-ranks testdemonstrated a significant change before (mdn = 3) and after using the D-Crastinate method (mdn = 2): Z = −4.08, *p* < 0.001.

In escapism type, people procrastinating to distance themselves from real-life issues. The result of the Wilcoxon signed-ranks testdemonstrated a significant change before (mdn = 3) and after (mdn = 2) using the D-Crastinate method: Z = −3.34, *p* = 0.001.

In the emergence type, people procrastinate on SNS due to the distracting nature of notifications. The result of the Wilcoxon signed-ranks test demonstrated a significant change, before (mdn = 4) and after using D-Crastinate (mdn = 2): Z = −4.03, *p* < 0.001.

In the second part, paired sample t-tests were conducted to examine the changes in respondents’ experience of procrastination triggers. The set of triggers includes notifications; surveillance of presence; identity; interaction; immersive design features. Table 9 presented the results of the features that led the participants to procrastination before and after using D-Crastinate method. Furthermore, the result of the Wilcoxon signed-ranks test is presented for each feature in the following subsections.

Concerning notification features, the results showed a significant change in experiencing notification-triggered procrastination before (mdn = 3) and after using D-Crastinate (mdn = 2): Z = −4.00, *p* < 0.001. Regarding surveillance of presence features, the result showed a significant change before (mdn = 2) and after using D-Crastinate (mdn = 1): Z = −4.05, *p* < 0.001. Concerning the procrastination triggered by identity features of SNS, the result showed a significant change before (mdn = 2) and after using the D-Crastinate method (mdn = 1): Z = −3.90, *p* < 0.001. Concerning the procrastination triggered by the interaction features of SNS, the result showed a significant change before (mdn = 2) and after (mdn = 1) using D-Crastinate: Z = −3.52, *p* < 0.001. Concerning the experience of procrastination triggered by the immersive design of SNS, the result showed a significant change before (mdn = 3.5) and after using the D-Crastinate method (mdn = 2): Z = −3.71, *p* < 0.001.

#### 4.2.5. Acceptance of D-Crastinate

We used “The e-Therapy Attitudes and Process Questionnaire (e-TAP)” to measure the acceptance of the D-Crastinate method and the extent to which the participants agreed to use this method in the future [57]. This questionnaire is built based on the theory of planned behaviour and meant to measure four components; behaviour intention; attitude toward behaviour; subjective norm; perceived behaviour control [59]. The combination of these components can predict the possibility of carrying out such a method in future; in this case, D-Crastinate. Planned behaviour theory is widely used to predict and measure people’s intention toward behavioural change; examples of studies that use planned behaviour theory scales are [60,61,62].

Table 10 presents the distribution of means and standard deviations for behaviour intention; attitude toward behaviour; subjective norm; perceived control of behaviour. A Shapiro-Wilk normality test determined that the scores on the components of e-TAP were normally distributed. The possible score range for the four subscales was 4–35; while the actual mean score for behaviour intention was 22.1 (SD = 3.1), which indicates that the participants had high intention to use D-Crastinate in the future. The participants had a highly favourable attitude toward using the D-Crastinate method in future, as evidenced by their mean score of 23.3 (SD = 2.8). For the subjective norm and perceived behavioural control subscales, the participants’ mean scores were 21.1 (SD = 4.0) and 23.4 (SD = 3.0), respectively. Therefore, the previous results demonstrated that the participants have positive attitudes toward using the D-Crastinate method in future.

## 5. Discussion

Concerning the experience of the different types of procrastination, seven respondents (23%) selected just one type of procrastination, but the majority of participants (18; 60%) selected two types. Meanwhile, one participant selected three types of procrastination and four (13%) participants selected all the procrastination types. It should be noted that none of the participants suggested a new type of procrastination. These results mean that the four procrastination types are comprehensive and represented most people’s procrastination experiences. Table 11 shows the number of the participants and their selected procrastination types; the most commonly selected type was ‘avoidance’, with 23 (76%) participants choosing it; ‘mood modification’ by 17 (56%); ‘emergence’ by 13 (43%) and ‘escapism’ by seven (23%). The participants reported that merely identifying the types of procrastination was helpful in reducing the possibility of procrastination. One participant said, “Now I understand why I usually procrastinate and this was very helpful to control my procrastination better”.

Regarding the SNS features that may trigger procrastination, 20 (67%) of the participants selected two features; seven (23%) selected one feature; one participant selected three features and two (7%) selected four features. The majority of the participants (18; 60%) selected ‘notifications’; ‘interaction’ was selected by 13 (43%); 11 (37%) chose ‘immersive design’; ‘surveillance of presence’ was selected by six (20%) and two (75%) selected ‘identity features’ (see Table 11). Increasing users’ awareness of the features that may lead them to procrastinate can play an important role in reducing procrastination time, while it also helped to select the appropriate countermeasures for each feature. Participants agreed that D-Crastinate method provided sufficient information about how SNS features could trigger procrastination. One participant said, “The examples provided for each feature helped me a great deal to better understand how the features trigger my procrastination”.

Concerning task engagement tools, 21 (70%) of the participants selected reduction tools and 20 (67%) selected reward, while 10 (33%) of the participants selected the task commitments tool (see Table 11). During the focus group session that was conducted after using D-Crastinate, most of the participants agreed that the main motivation for their procrastination was lack of motivation, combined with demanding tasks that required a long time to finish. One participant said, “Breaking a huge task into smaller tasks has really helped me to achieve them”.

Concerning procrastination countermeasures, ‘time restriction’ was most chosen by most people (13; 43%); 10 (33%) selected ‘suggestion’; ‘chat timer’ was selected by eight (26%); eight (26%) chose ‘showing availability’; six (20%) chose ‘task priority’, ‘usage reminder’ and ‘auto-reply’; two participants selected ‘usage feedback’; ‘goal setting’ and ‘reminder for both users’ were selected one time each (see Table 11). The majority (26; 87%) of the participants reported that the selected countermeasures had worked for them from the first week of use and were easy to understand and apply. However, four (13%) participants reported that the countermeasures did not work well enough from the first time of use. The D-Crastinate method overcomes this possibility by adding an error identification stage to guide those people toward the appropriate selection. For example, one participant said, “It was interesting to see that one way worked and the other did not when I used another countermeasure”.

Since some of the countermeasures do not yet exist, the participants had to apply them manually or use external tools. This may suggest why only two participants chose ‘reminder for both users’ and ‘goal setting’. To overcome this issue, the evaluation study provided alternative countermeasures that relate closely to the countermeasures that are still to be offered by the de-facto SNS and tools. Some participants noted that one week was a relatively short time to evaluate the effectiveness of the countermeasures, feeling that they needed more time to live and feel the countermeasures. However, the aim of the evaluation was to examine whether the D-Crastinate method has the potential to improve the participants’ control over their procrastination. Sustaining that change and relapse prevention require additional stages and tools to support.

### Limitations

In the course of a research project, several factors might affect the validity of the study. One of the ways for accessing these threats to validity is by grouping them into internal and external threats. Internal threats to validity refer to the study actually carried out with the participants, and whether it was carried out in a way that makes the results accurate. External threats to validity refer to the extent to which the results of the study can be generalised [63].

The internal threats to validity in this study include the time allowed for testing, the subjectivity of participants and the feasibility of some of the countermeasures proposed. The study was carried out over a two-week period. This period might not, however, be sufficient to determine significant changes in social media usage and procrastination. To determine whether the change in procrastination behaviour was significant, participants would ideally be subjected to test conditions for a much longer period. Thus, this research was only able to test whether participants benefited and if they felt there had been improvements. The mission of D-Crastinate it to trigger a behaviour change in a way that is acceptable and easy to follow by the subjects. Nevertheless, there is evidence from other domains, such as excessive alcohol use, that brief interventions can result in long-term behaviour change [64]. As research into strategies to reduce SNS facilitated procrastination continues, the optimal intervention duration can be better identified. We also note that D-Crastinate can be enhanced to tailor a staged approach to the application of countermeasures, e.g., advising to get used to setting a status or configuring an auto-reply in the first weeks before recommending the application of more advanced measures such as expectation management and conversation protocols. This is consistent with goal setting best practice in seeking achievable and realistic goals as well as the persuasion and influence principle of reduction [65,66]. In our future work, we will seek to refine D-Crastinate with recommendations on how the countermeasures can be applied in a gradual and phased style.

We note that the questionnaire that was developed in this study did not undergo tests of reliability or validity. We developed our own questionnaire due to the lack of existing, suitable measures. The questionnaire was in part intended to facilitate the contemplation stage of the behaviour change process, in keeping with the transtheoretical model [53]. As such, this questionnaire is not intended to function as a clinical measure of procrastination. Given the relatively small sample size we also acknowledge that the inferential analysis on the questionnaire responses before and after completing the D-Crastinate method should be treated with caution.

Furthermore, the subjectivity of participants may be a threat to validity in a study of this nature. For instance, the perception of procrastination could differ depending on the period of the year. Thus, what students perceive as procrastination during exam periods might not be perceived as such during the summer. Therefore, testing for improvements might not be as straightforward as expected, as seasonal changes might affect participants’ perception of procrastination. This subjectivity of participants can also extend to how they perceive and interpret their actions as improvements, which affects the overall research findings. As the study continued for two weeks at the start of the academic semester, there were no major changes in the work environment during the study, and this minimised this risk.

Another internal threat to validity is the countermeasures tested in the course of this study. Some proposed countermeasures have not yet been implemented in SNS and thus cannot be currently tested. These countermeasures include suggestions and chat timers. To overcome this threat, several alternative techniques were proposed to help participants stimulate how these countermeasures could control procrastination on SNS in the future. Some required manual practice and others can be supported by external tools e.g., for time limits and muting notification.

The external threats to validity in this study mainly come from the sample population utilised, and the non-specificity of social media networks analysed. The study participants were mainly university students. While this is not an issue in itself, as students face similar procrastination issues as other members of society, it could be argued that this sample population is a user group that is educated and tech-savvy. Thus, the generalisation of the findings to less educated or tech-savvy groups might be problematic. The sample population of 30 participants is not enough to reveal culture-related differences. Peer pressures and social norms differ across cultures, and these are the main reasons that influence the urge to respond quickly, be online, and eventually procrastinate.

Furthermore, our evaluation did not focus on a specific SNS platform, and it might be the case that procrastination and countermeasures are platform-specific. For instance, the procrastination pattern on Snapchat and its countermeasures might be different from those of WhatsApp. Snapchat users might procrastinate due to the fear of missing temporary content, as uploaded media are only available for 24 h. Snapchat users might therefore need more specific countermeasures, such as suggestions. In contrast, WhatsApp users might procrastinate due to the pressure to respond instantly when they receive a message, especially when they are visible online. Thus, showing availability countermeasures could be used in this case to reduce such pressure and eventually avoid procrastination. Hence, the results, despite being in favour of D-Crastinate, did not enable us to study whether they do apply at the same level amongst the different SNS types.

## 6. Conclusions

In this paper, we presented D-Crastinate, which aims to help users to gain greater control over their procrastination on SNS. We built the method based on a set of psychological theories, and our previous research result in [8,12,13]. This paper also discussed the evaluation process of D-Crastinate. In the evaluation study, we adopted a mixed-methods approach which included a focus group, diary study (qualitative) and survey (quantitative) to examine the extent to which the participants believed that D-Crastinate offered a useful and effective method to control procrastination on SNS. The results indicated the possible usefulness of D-Crastinate in reducing the tendency for procrastination. Additionally, the participants exhibited a positive attitude regarding the use of D-Crastinate in future. The results support an argument that SNS should integrate education and tools to combat procrastination into their design. Such integration can also benefit from automated monitoring of usage data and minimise the reliance on self-report. Further integration to other sources of data, e.g., calendar and contacts, will also help drawing a more accurate picture of procrastination and its context. The study is meant as a first step and to provide indicators of the need for methods to assist in regulating procrastination on social media. We demonstrated acceptance for our proposed method, D-Crastinate, through an initial study and a potential for it to help awareness of both procrastination and its countermeasures. The long-term effect of the method and relapse prevention shall require additional stages and tools, and we will focus on that in our future work.

## Figures and Tables

**Table 1:** Stages of D-Crastinate method and their expected activities and outcomes.

Method Stages	Expected Activity and Outcomes
First stage: Education	Increase users’ awareness about procrastination and how it happens in general Build digital resilience through enabling users to understand their motivation for procrastination and how to combat it Users get knowledge about relapse prevention
Second stage: Self-diagnosis	Users identify their procrastination types. Users identify the features of SNS that facilitate their procrastination.
Third stage: Planning and preparation	Users identify the tools that increase their engagement in the tasks they typically avoid or delay Users identify the suitable technical and socio-technical countermeasures to combat procrastination.
Fourth stage: Action	Users use the customised countermeasures for a period of time (typically one week). Alternative countermeasures are made available when the suggested countermeasures do not work well enough.
Fifth stage: Self-assessment	Users assess the usefulness of the suggested method and whether it helps to combat procrastination.
Sixth stage: Error identification	Users identify the features of SNS and the countermeasures more specifically should those identified in previous stages were deemed to be insufficient.

**Table 2:** Guidance for applying D-Crastinate method.

Stage’s Name	Guidance
Education	In the education stage, users are offered material to read and familiarise themselves with the key ideas that may lead people to procrastinate more on SNS (see pages 1 to 8 in the Booklet provided as a supplementary material of this paper).
Self-diagnosis	Firstly, in this stage, D-Crastinate helps users to figure out what type of procrastinator they are (see Page 9 in the Booklet). Procrastination types are avoidance, mood modification, escapism, and emergence (see Table 3). Secondly, users can determine for themselves the main features of social networking sites that facilitate their procrastination (see Page 10 in the Booklet). These features are notification, immersive design, surveillance of presence, identity, interaction (see Table 4). However, if users feel like they procrastinate due to other motives that are not currently listed, they may write them down before moving on to the planning and preparation stage.
Planning and preparation	Firstly, if users believe that they procrastinate due to a lack of motivation to complete their tasks, they can consider using tasks engagement tools (see Page 11 in the Booklet). These tools include reward, reduction, and task commitment (see Table 5).Secondly, we provide a list of customised countermeasures that can help users to gain more control over how much they procrastinate on a day-to-day basis (see Pages 12 to 14 in the booklet). The countermeasures have been paired with the features that lead them to procrastinate (see Table 4).
Action	In this stage, users are required to apply the selected tools of tasks engagement and the countermeasures for one week (see Page 15 in the Booklet).
Self-assessment	After the action stage is completed, users are expected to decide how useful they found the previous stages (see Table 6 and Page 16 in the Booklet). However, users can move on to the next stage if they do not find the previous stages useful in helping them to gain more control over their procrastination.
Error identification	In this stage, users are expected to answer the provided questions to help them analyse what went wrong in the previous stages (see Page 17 in the Booklet). Once users have identified their challenges, they can then return to the second stage to apply the method process again.

**Table 3:** Procrastination types.

Procrastination Types	Question
Avoidance	I often procrastinate to avoid working on unpleasant or difficult tasks
Mood modification	I often procrastinate to change my mood and feel better
Escapism	I often procrastinate to distance myself from real-life issues
Emergence	When I receive a notification, I check it and spend time on that despite having other tasks to perform

**Table 4:** Social networking site (SNS) Features as procrastination triggers and their suggested countermeasures.

SNS Features as Procrastination Triggers	Question	Suggested Countermeasures	Example
Notification features	I often delay working on my tasks because I am busy checking notifications on social media	Auto-reply	e.g., sending an auto-reply that contains some information such as I received your message, I will read and reply later when finishing my current work.
		Showing availability	e.g., when you receive a notification, your contacts are informed that you are unavailable or busy
		Suggestions	e.g., at the same time as the notification, you receive a message suggesting how to avoid procrastination, e.g., showing how to mute notification and how to declare a Busy status.
Immersive design features	On social media, I spend time more than I initially intended due to seeing relevant content suggested to me automatically	Time restriction	e.g., restricting you from using social media beyond a maximum time or during certain hours of the day that you sat for yourself.
		Usage reminder	e.g., when you decide to spend 30 min on social media, you receive a reminder about the time that you have spent once you approach that limit.
		Usage feedback	e.g., at the end of the day, you can see statistics regarding the time you spent on social media and when such a usage conflicted with your other tasks listed in your online calendar.
Surveillance of presence features	When I send a message to someone, I keep checking whether they received, read or replied my message	Auto-reply	e.g., receiving an automated message from your contacts containing information such as I am currently busy and will try to read and reply when I am free around 5:00 pm today.
		Task Priority	e.g., showing you your priority tasks and to-do list so that you focus on them and avoid unnecessary checking.
Identity features	I procrastinate on social media to maintain a positive image and interaction with people and respond to them on a timely fashion	Usage feedback	e.g., at the end of the day, you can see statistics regarding the time you spent on social media and when such a usage is conflicted with other tasks listed in your online calendar.
		Time restriction	e.g., restricting you from using social media beyond a maximum time or during certain hours of the day that you sat for yourself.
		Auto-reply	e.g., sending an automated message to your contacts containing information such as: I am currently busy and will try to read and reply when I am free around 5:00pm today.
		Goal setting	e.g., enabling you to set your career or life-related goals, and help you to track your progress toward achieving these goals.
Interaction features	When I am involved in chatting, I find it hard to stop procrastinating and complete my tasks	Reminder to both users	e.g., while chatting, both of you receive a reminder telling that one or both of you may have other work to do as your online calendar suggests.
		Showing availability	e.g., your status will automatically change and declare that you have now become busy with other tasks so your friends would not expect you to continue chatting.
		Chatting timer	e.g., a time bar showing both users the time limit for the chat and the time spent already.

**Table 5:** Task engagement tools.

Engagement Tools	Question
Rewards	I am more motivated to work on tasks that have rewards such as virtual points for each accomplished level and performance quality.
Reduction	I would like to specify different milestones for my big tasks and have a deadline for each milestone.
Task commitments	Declaring my work commitments to my contacts on social media would help me to commit more to fulfil them and reduce the peer pressure to engage in unnecessary conversations.

**Table 6:** Self-assessment’s question.

Self-Assessment’s Question	Yes	No
Do the suggested countermeasures help you to gain more control over your procrastination?		

**Table 7:** Comparison of participants’ awareness before and after using D-Crastinate.

**Questions**		**Yes**	**No**		**Not Sure**	
Do you know how procrastination on social networking sites happens?	Before	33.3%	23.3%		43.3%	
	After	96.7%	0%		3.3%	
**Questions**		**Extremely Aware**	**Moderately Aware**	**Somewhat Aware**	**Slightly Aware**	**Not at All**
Are you aware of the features that may facilitate procrastination on social networking sites?	Before	0%	30%	33.3%	20%	16.7%
	After	40%	53.3%	6.7%	0%	0%
How do you rate your awareness of how to control your procrastination on social networking sites?	Before	0%	20%	46.7%	23.3%	10%
	After	40%	46.7%	13.3%	0%	0%

**Table 8:** Experiencing procrastination in its various types before and after using D-Crastinate.

Procrastination Types		Never	Rarely	Sometimes	Often	Always
Avoidance	Before	0%	10%	13.3%	43.3	33.3%
	After	0%	36.7%	50%	6.7%	6.7%
Mood modification	Before	3.3%	20%	33.3%	16.7%	26.7%
	After	20%	53.3%	20%	6.7%	0%
Escapism	Before	23.3%	16.7%	23.3%	26.7%	10%
	After	46.7%	26.7%	23.3%	3.3%	0%
Emergence	Before	3.3%	3.3%	30%	46.7%	16.7%
	After	20%	40%	33.3%	6.7%	0%

**Table 9:** Experiencing procrastination due to SNS triggers before and after using D-Crastinate method.

SNS Features		Never	Rarely	Sometimes	Often	Always
Notification	Before	3.3%	13.3%	40%	30%	13.3%
	After	16.7%	60%	16.7%	3.3%	3.3%
Surveillance of presence	Before	13.3%	43.3%	20%	13.3%	10%
	After	53.3%	36.7%	10	0%	0%
Identity	Before	16.7%	46.7%	20%	10%	6.7%
	After	53.3%	40%	6.7%	0%	0%
Interaction	Before	3.3%	26.7%	40%	23.3%	6.7%
	After	13.3%	60%	23.3%	3.3%	0%
Immersive design	Before	0%	20%	30%	33.3%	16.7%
	After	26.7%	36.7%	33.3%	0%	3.3%

**Table 10:** Distribution of means and standard deviations for e-TAP components.

PBT Components	N	Possible Range	Mean	Std. Deviation
Behaviour intention	30	4–35	22.1	3.1
Attitude toward behaviour	30	4–35	23.3	2.8
Subjective norm	30	4–35	21.1	4.0
Perceived behaviour control	30	4–35	23.4	3.0

**Table 11:** Participants’ selection for procrastination types, triggers, and countermeasures.

**Procrastination types**				
Avoidance	Mood modification	Emergence	Escapism	
23	17	13	7	
**SNS features that may facilitate procrastination**				
Notifications	Immersive design	Interaction	Identity	Surveillance of presence
18	11	13	2	6
**Procrastination countermeasures**				
Time restriction	Suggestion	Task priority	Usage reminder	Chat timer
13	10	6	6	8
Usage feedback	Goal setting	Auto reply	Showing availability	Reminder for both users
2	1	6	7	1
**Task engagement tools**				
Reduction		Reward	Tasks commitment	
21		20	10	

## Supplementary Materials

The following are available online at https://www.mdpi.com/2227-9032/8/4/577/s1, supplementary S1 Combating Procrastination on Social Networking Sites (D-Crastinate).

## Appendix A. Questionnaire before Using the D-Crastinate Method

Q1/I often procrastinate on social networking sites instead of working on my tasks

◯   Yes ◯   No (if your answer is no, please stop the survey here)

Q2/What is your age?

Q3/What gender do you identify with? ◯    Male ◯   Female ◯   I prefer not to say

Q4/Do you know how procrastination on social networking sites happens? If yes, please elaborate on your answer

◯   Yes ◯   No ◯   Not sure

Q5/Are you aware of the features that may facilitate procrastination on social networking sites?

◯   Extremely aware ◯   Moderately aware ◯   Somewhat aware ◯   Slightly aware ◯   Not at all

Q6/How do you rate your awareness of how to control your procrastination on social networking sites?

◯   Extremely aware ◯   Moderately aware ◯   Somewhat aware ◯   Slightly aware ◯   Not at all

Q7/[Using the scale of (never, rarely, sometimes, often, always] Please tick the word that most closely represents your experience of procrastination on social networking sites

- I often procrastinate to avoid working on unpleasant or difficult tasks
- I often procrastinate to change my mood and feel better
- I often procrastinate to distance myself from real-life issues
- When I receive a notification, I check it and spend time on that, despite having other tasks to perform

Q8/[Using the scale of (never, rarely, sometimes, often, always)] Please tick the word that most closely represents your experience of procrastination on social media in the following sentences.

- I often get distracted by notifications which lead me to access social networking sites and then delay working on my tasks.
- When I send a message to someone, I keep checking whether or not they are active online?
- I respond to my contacts on social media almost instantly in order to build a positive self-image and maintain a good profile.
- I find it hard to pull myself away from online conversations in order to complete my tasks.
- While on social media, I often see suggested content that is relevant to me and I end up spending more time than I intended to spend on those sites.

## Appendix B. Questions after Using D-Crastinate Method

Q1/Do you know how procrastination on social networking sites happens? If yes, please elaborate on your answer.

◯    Yes ◯    No ◯    Not sure

Q2/Are you aware of the features that may facilitate procrastination on social networking sites?

◯   Extremely aware ◯   Moderately aware ◯   Somewhat aware ◯   Slightly aware ◯   Not at all

Q3/How do you rate your awareness of how to control your procrastination on social networking sites?

◯   Extremely aware ◯   Moderately aware ◯   Somewhat aware ◯   Slightly aware ◯   Not at all

Q4/[Using the scale of (never, rarely, sometimes, often, always)] Please tick the word that most closely represents your experience of procrastination on social media in the last week

- I often procrastinate to avoid working on unpleasant or difficult tasks
- I often procrastinate to change my mood and feel better
- I often procrastinate to distance myself from real-life issues
- When I receive a notification, I check it and spend time on that, despite having other tasks to perform

Q5/[Using scale of (never, rarely, sometimes, often, always)] Please tick the box that most closely represents your experience of procrastination on social media in the last week

- often get distracted by notifications which lead me to access social networking sites and then delay working on my tasks.
- When I send a message to someone, I keep checking whether or not they are active online?
- I respond to my contacts on social media almost instantly in order to build a positive self-image and maintain a good profile.
- I find it hard to pull myself away from online conversations in order to complete my tasks.
- While on social media, I often see suggested content that is relevant to me and I end up spending more time than I intended to spend on those sites.

Q6: Sufficient information was provided regarding how to use the D-Crastinate method:

◯   Strongly Agree ◯   Agree ◯   Neutral ◯   Disagree ◯   Strongly Disagree

Q7: Sufficient information was provided for the D-Crastinate method about the types of procrastination:

◯   Strongly Agree ◯   Agree ◯   Neutral ◯   Disagree ◯   Strongly Disagree

Please elaborate on your answer (optional):

Q8: Sufficient information was provided for the D-Crastinate method about the features of social networking sites that lead to procrastination:

◯   Strongly Agree ◯   Agree ◯   Neutral ◯   Disagree ◯   Strongly Disagree

Please elaborate on your answer (optional):

Q9: Sufficient information was provided for the D-Crastinate method about task engagement tools:

◯   Strongly Agree ◯   Agree ◯   Neutral ◯   Disagree ◯   Strongly Disagree

Please elaborate on your answer (optional):

Q10: Sufficient information was provided for the D-Crastinate method about the countermeasures for combating procrastination:

◯   Strongly Agree ◯   Agree ◯   Neutral ◯   Disagree ◯   Strongly Disagree

Please elaborate on your answer (optional):

Q11: Generally speaking, the D-Crastinate method was not difficult to understand (e.g., it was explained in a clear way):

◯   Strongly Agree ◯   Agree ◯   Neutral ◯   Disagree ◯   Strongly Disagree

Please elaborate on your answer (optional):

Q12: Overall, the D-Crastinate method was not difficult to use:

◯   Strongly Agree ◯   Agree ◯   Neutral ◯   Disagree ◯   Strongly Disagree

Please elaborate on your answer (optional):

Q13: Did you encounter issues or difficulties when using the D-Crastinate method? If yes, please explain:

Q14: Did you experience any habitual behaviour while you were applying D-Crastinate? If yes, please explain:

## Appendix C. The e-Therapy Attitudes and Process Questionnaire (eTAP)

Please circle the number that most closely represents your experience when using the D-Crastinate method for procrastination

- I will use the D-Crastinate method to gain better control over my procrastination on SNS in the next week:(1 (Strongly disagree), 2, 3, 4, 5, 6, and 7 (Strongly agree)).
- I find the D-Crastinate method for controlling procrastination on SNS to be: (−3 (Unhelpful), −2, −1, 0, 1, 2, and 3 (Helpful)).
- Those people who are important to me would approve of me using the D-Crastinate method to gain a better control over my procrastination on SNS: (1 (Strongly disagree), 2, 3, 4, 5, 6, and 7 (Strongly agree)).
- I possess the required knowledge to use the D-Crastinate method to gain better control over my procrastination on SNS:(1 (Strongly disagree), 2, 3, 4, 5, 6, and 7 (Strongly agree)).
- It is likely that I will use the D-Crastinate method to gain better control over my procrastination on SNS in the next week:(1 (Strongly disagree), 2, 3, 4, 5, 6, and 7 (Strongly agree)).
- Most people who are important to me would approve of me using the D-Crastinate method to gain better control over my procrastination on SNS:(1 (Strongly disagree), 2, 3, 4, 5, 6, and 7 (Strongly agree)).
- I find using the D-Crastinate method to gain better control over my procrastination on SNS to be: (−3 (Harmful), −2, −1, 0, 1, 2, and 3 (Beneficial)).
- It is mostly up to me whether I use the D-Crastinate method to gain better control over my procrastination on SNS in the next week: (1 (Strongly disagree), 2, 3, 4, 5, 6, and 7 (Strongly agree)).
- I intend to use the D-Crastinate method to gain better control over my procrastination on SNS in the next week: (1 (Strongly disagree), 2, 3, 4, 5, 6, and 7 (Strongly agree)).
- I find using the D-Crastinate method to gain better control over my procrastination on SNS to be: (−3 (Unpleasant), −2, −1, 0, 1, 2, and 3 (Pleasant)).
- Those people who are important to me would support me using the D-Crastinate method to gain better control over my procrastination on SNS: (1 (Strongly disagree), 2, 3, 4, 5, 6, and 7 (Strongly agree)).
- I intend to ensure that I have access to the required materials to use the D-Crastinate method to gain better control over my procrastination on SNS in the next week: (1 (Strongly disagree), 2, 3, 4, 5, 6, and 7 (Strongly agree)).
- I have complete control over whether I use the D-Crastinate method to gain better control over my procrastination on SNS: (1 (Strongly disagree), 2, 3, 4, 5, 6, and 7 (Strongly agree)).
- I find the D-Crastinate method to offer better control over my procrastination on SNS to be: (-3 (Not credible), −2, −1, 0, 1, 2, and 3 (Credible)).
- I am confident using the D-Crastinate method to gain better control over my procrastination on SNS: (1 (Strongly disagree), 2, 3, 4, 5, 6, and 7 (Strongly agree)).
- Those people who are important to me think that the D-Crastinate method for procrastination on SNS is credible: (1 (Strongly disagree), 2, 3, 4, 5, 6, and 7 (Strongly agree).

## References

[1] Klingsieck K.B. (2013). Procrastination: When good things don’t come to those who wait. Eur. Psychol..

[2] Knaus W.J. (1973). Overcoming procrastination. Ration. Living.

[3] Steel P. (2007). The nature of procrastination: A meta-analytic and theoretical review of quintessential self-regulatory failure. Psychol. Bull..

[4] Beheshtifar M., Hoseinifar H., Moghadam M. (2011). Effect procrastination on work-related stress. Eur. J. Econ. Financ. Adm. Sci..

[5] Vitak J., Crouse J., LaRose R. (2011). Personal Internet use at work: Understanding cyberslacking. Comput. Hum. Behav..

[6] Cao X., Yu L. (2019). Exploring the influence of excessive social media use at work: A three-dimension usage perspective. Int. J. Inf. Manag..

[7] Alutaybi A., Arden-Close E., McAlaney J., Stefanidis A., Phalp K., Ali R. How Can Social Networks Design Trigger Fear of Missing Out?. Proceedings of the IEEE International Conference on Systems, Man and Cybernetics (SMC).

[8] Alblwi A., Stefanidis A., Phalp K., Ali R. Procrastination on Social Networks: Types and Triggers. Proceedings of the 6th International Conference on Behavioral Economic, and Socio-Cultural Computing.

[9] Schouwenburg H.C., Lay C.H. (1995). Trait procrastination and the Big-five factors of personality. Pers. Individ. Differ..

[10] Kuss J.D., Griffiths D.M., Karila L., Billieux J. (2014). Internet addiction: A systematic review of epidemiological research for the last decade. Curr. Pharm. Des..

[11] Reinecke L., Hofmann W. (2016). Slacking off or winding down? An experience sampling study on the drivers and consequences of media use for recovery versus procrastination. Hum. Commun. Res..

[12] Alblwi A., Stefanidis A., Phalp K., Ali R. Procrastination on Social Networking Sites: Combating by Design. Proceedings of the 2019 13th International Conference on Research Challenges in Information Science (RCIS).

[13] Alblwi A., McAlaney J., Altuwairiqi M., Stefanidis A., Phalp K., Ali R. (2020). Procrastination on Social Networks: Triggers and Countermeasures. Psihologija.

[14] Borsari B., Carey K.B. (2003). Descriptive and injunctive norms in college drinking: A meta-analytic integration. J. Stud. Alcohol.

[15] Borsari B., Carey K.B. (2001). Peer influences on college drinking: A review of the research. J. Subst. Abus..

[16] McAlaney J., Bewick B., Hughes C. (2011). The international development of the ‘Social Norms’ approach to drug education and prevention. Drugs Educ. Prev. Policy.

[17] Perkins H.W. (2002). Social norms and the prevention of alcohol misuse in collegiate contexts. J. Stud. Alcohol Suppl..

[18] Croker H., Whitaker K., Cooke L., Wardle J. (2009). Do social norms affect intended food choice?. Prev. Med..

[19] Stok F.M., De Ridder D.T., De Vet E., De Wit J.B. (2012). Minority talks: The influence of descriptive social norms on fruit intake. Psychol. Health.

[20] Alutaybi A., McAlaney J., Stefanidis A., Phalp K., Ali R. (2018). Designing Social Networks to Combat Fear of Missing Out. Proceedings of the HCI 2018.

[21] Hoadley C.M., Xu H., Lee J.J., Rosson M.B. (2010). Privacy as information access and illusory control: The case of the Facebook News Feed privacy outcry. Electron. Commer. Res. Appl..

[22] Van Dyke T.P., Midha V., Nemati H. (2007). The effect of consumer privacy empowerment on trust and privacy concerns in e-commerce. Electron. Mark..

[23] Moeller F.G., Barratt E.S., Dougherty D.M., Schmitz J.M., Swann A.C. (2001). Psychiatric aspects of impulsivity. Am. J. Psychiatry.

[24] Barratt E.S. (1959). Anxiety and impulsiveness related to psychomotor efficiency. Percept. Mot. Ski..

[25] Barratt E.S. (1970). Anxiety and Impulsiveness: Toward a Neuropsychological Model.

[26] Gustavson D.E., Miyake A., Hewitt J.K., Friedman N.P. (2014). Genetic relations among procrastination, impulsivity, and goal-management ability: Implications for the evolutionary origin of procrastination. Psychol. Sci..

[27] Sirois F., Pychyl T. (2013). Procrastination and the priority of short-term mood regulation: Consequences for future self. Soc. Personal. Psychol. Compass.

[28] Eckert M., Ebert D.D., Lehr D., Sieland B., Berking M. (2016). Overcome procrastination: Enhancing emotion regulation skills reduce procrastination. Learn. Individ. Differ..

[29] De Paola M., Scoppa V. (2015). Procrastination, academic success and the effectiveness of a remedial program. J. Econ. Behav. Organ..

[30] Tice D.M., Bratslavsky E., Baumeister R.F. (2001). Emotional distress regulation takes precedence over impulse control: If you feel bad, do it!. J. Personal. Soc. Psychol..

[31] Lakein A., Leake P. (1973). How to Get Control of Your Time and Your Life.

[32] Slaven G., Totterdell P. (1993). Time management training: Does it transfer to the workplace?. J. Manag. Psychol..

[33] Francis-Smythe J.A., Robertson I.T. (1999). On the relationship between time management and time estimation. Br. J. Psychol..

[34] Van Eerde W. (2015). Time management and procrastination. Psychol. Plan. Organ. Res. Appl..

[35] LaRose R. (2010). The problem of media habits. Commun. Theory.

[36] LaRose R., Eastin M.S. (2004). A social cognitive theory of Internet uses and gratifications: Toward a new model of media attendance. J. Broadcast. Electron. Media.

[37] Kaye B.K. (1998). Uses and gratifications of the World Wide Web: From couch potato to Web potato. Atl. J. Commun..

[38] Ferguson D.A., Perse E.M. (2000). The World Wide Web as a functional alternative to television. J. Broadcasting Electron. Media.

[39] Song I., Larose R., Eastin M.S., Lin C.A. (2004). Internet gratifications and Internet addiction: On the uses and abuses of new media. Cyberpsychology Behav..

[40] Chou C., Hsiao M.-C. (2000). Internet addiction, usage, gratification, and pleasure experience: The Taiwan college students’ case. Comput. Educ..

[41] Chen H.-T., Kim Y. (2013). Problematic use of social network sites: The interactive relationship between gratifications sought and privacy concerns. Cyberpsychology Behav. Soc. Netw..

[42] Boyle K., Johnson T.J. (2010). MySpace is your space? Examining self-presentation of MySpace users. Comput. Hum. Behav..

[43] Janz N.K., Becker M.H. (1984). The health belief model: A decade later. Health Educ. Q..

[44] Marlatt G.A., Donovan D.M. (2005). Relapse Prevention: Maintenance Strategies in the Treatment of Addictive Behaviors.

[45] Larimer M.E., Marlatt G.A. (2004). Relapse prevention: An overview of Marlatt’s cognitive-behavioral model. Psychosocial Treatments.

[46] Brooks R., Goldstein H.S., Brooks R.B. (2005). The Power of Parenting. Handbook of Resilience in Children.

[47] Scheier M.F., Carver C.S. (1993). On the power of positive thinking: The benefits of being optimistic. Curr. Dir. Psychol. Sci..

[48] Ryan R.M., Deci E.L. (2000). Self-determination theory and the facilitation of intrinsic motivation, social development, and well-being. Am. Psychol..

[49] Shafran R., Mansell W. (2001). Perfectionism and psychopathology: A review of research and treatment. Clin. Psychol. Rev..

[50] Frost R.O., Marten P., Lahart C., Rosenblate R. (1990). The dimensions of perfectionism. Cogn. Ther. Res..

[51] Lundh L.-G. (2004). Perfectionism and acceptance. J. Ration. Emot. Cogn. Behav. Ther..

[52] Prochaska J.O., DiClemente C.C. (1983). Stages and processes of self-change of smoking: Toward an integrative model of change. J. Consult. Clin. Psychol..

[53] Krebs P., Norcross J.C., Nicholson J.M., Prochaska J.O. (2018). Stages of change and psychotherapy outcomes: A review and meta-analysis. J. Clin. Psychol..

[54] Seaman C.B. (1999). Qualitative methods in empirical studies of software engineering. IEEE Trans. Softw. Eng..

[55] Runeson P., Höst M. (2009). Guidelines for conducting and reporting case study research in software engineering. Empir. Softw. Eng..

[56] Kankainen A., Vaajakallio K., Kantola V., Mattelmäki T. (2012). Storytelling Group–A co-design method for service design. Behav. Inf. Technol..

[57] Clough B.A., Eigeland J.A., Madden I.R., Rowland D., Casey L.M. (2019). Development of the eTAP: A brief measure of attitudes and process in e-interventions for mental health. Internet Interv..

[58] Alblwi A. (2020). Procrastination on Social Networking Sites: Types, Triggers, and Socio-Technical Countermeasures. Ph.D. Thesis.

[59] Ajzen I. (1991). The theory of planned behavior. Organ. Behav. Hum. Decis. Process..

[60] Armitage C.J. (2005). Can the theory of planned behavior predict the maintenance of physical activity?. Health Psychol..

[61] Stolte E., Hopman-Rock M., Aartsen M.J., Van Tilburg T.G., Chorus A. (2017). The theory of planned behavior and physical activity change: Outcomes of the aging well and healthily intervention program for older adults. J. Aging Phys. Act..

[62] Judge M., Warren-Myers G., Paladino A. (2019). Using the theory of planned behaviour to predict intentions to purchase sustainable housing. J. Clean. Prod..

[63] Onwuegbuzie A.J., Leech N.L. (2007). Validity and qualitative research: An oxymoron?. Qual. Quant..

[64] Bertholet N., Daeppen J.B., Wietlisbach V., Fleming M., Burnand B. (2005). Reduction of alcohol consumption by brief alcohol intervention in primary care: Systematic review and meta-analysis. Arch. Intern. Med..

[65] Cham S., Algashami A., McAlaney J., Stefanidis A., Phalp K., Ali R. Goal Setting for Persuasive Information Systems: Five Reference Checklists. Proceedings of the International Conference on Persuasive Technology.

[66] Oinas-Kukkonen H., Harjumaa M. (2009). Persuasive systems design: Key issues, process model, and system features. Commun. Assoc. Inf. Syst..

