# Supplementary material for: Managing Procrastination on Social Networking Sites: The D-Crastinate Method
Source: Healthcare (Basel). 2020 Dec 18;8(4):577. doi: 10.3390/healthcare8040577 (PMC7766803; doi:10.3390/healthcare8040577)
Supplement: Supplementary file 1 [file healthcare-08-00577-s001.pdf]

Combating Procrastination on Social Networking Sites (D-Crastinate)

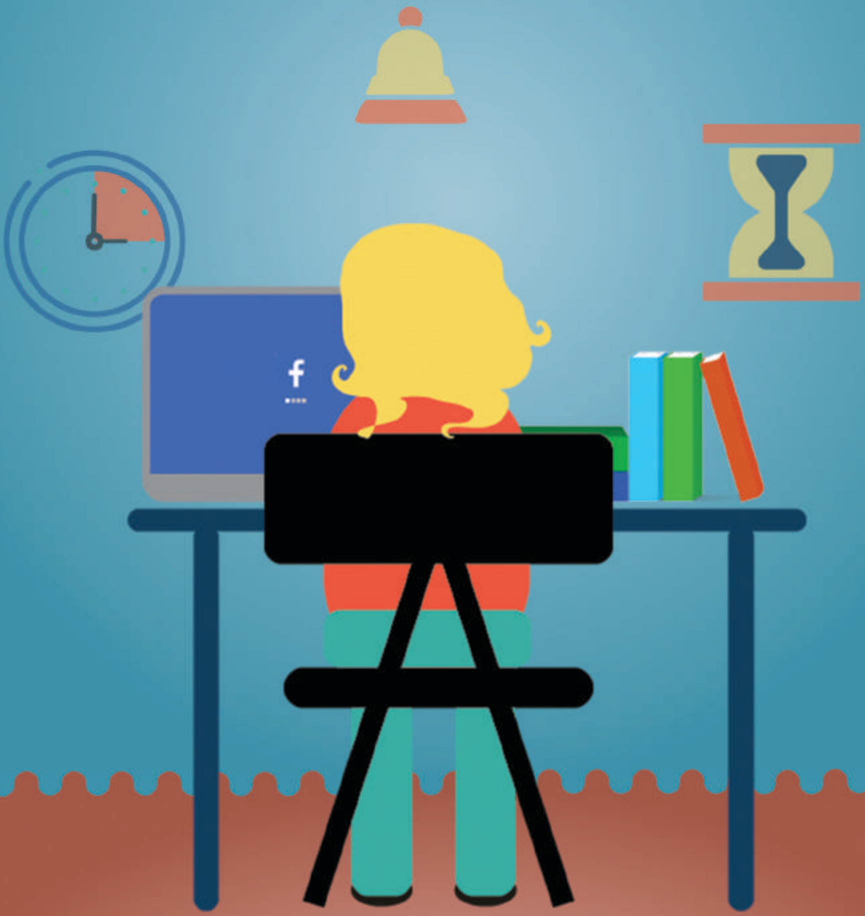

CONTENT PAGE

1 WHAT IS PROCRASTINATION?

2 D-Crastinate STAGES

3 GUIDANCE FOR USING THE D-Crastinate METHOD

4 FIRST STAGE: EDUCATION

5-6 HOW DOES PROCRASTINATION ON SOCIAL NETWORKING SITES HAPPEN?

7-8 SOLUTIONS FOR PROCRASTINATION

9 SECOND STAGE: SELF-DIAGNOSIS

10 SECOND STAGE: SELF-DIAGNOSIS

11 THIRD STAGE: PLANNING AND PREPARATION

12-14 THIRD STAGE: PLANNING AND PREPARATION

15 FOURTH STAGE: ACTION

16 FIFTH STAGE: SELF-ASSESSMENT

17 SIXTH STAGE: ERROR IDENTIFICATION

What is procrastination?

Procrastination is a voluntary delay, a way of avoiding tasks that need to be accomplished but which someone doesn't perhaps want to do.

When someone procrastinates too much it can lead to negative consequences.

Consequences

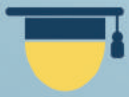

Low academic performance

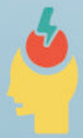

Anxiety

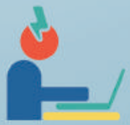

Work-related stress

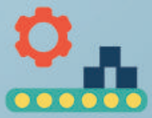

Low productivity

D-Crastinate Stages

The following table summarises the D-Crastinate method and its stages:

METHOD | STAGE NAME	FIRST STAGE<br>Education	SECOND STAGE<br>Self-diagnosis	THIRD STAGE<br>Planning and preparation	FOURTH STAGE<br>Action	FIFTH STAGE<br>Self-assesment	SIXTH STAGE<br>Error identification
EXPECTED OUTCOMES	• Increase your awareness about procrastination and how it happens in general.<br>• Help you to understand why you procrastinate.<br>• Help you to become more digitally resilient so you can better control your procrastination.<br>• Help you to gain more knowledge about what the relapse phase is, and how to prevent it.	• Help you find out your procrastination types.<br>• Help you to recognise the features of SNS that lead you to procrastinate more often.	• Aid you in finding useful tools that will help you to engage with particular tasks more.<br>• Teach you how to identify the suitable technical and socio-countermeasures to help you control your procrastination.	• You will use your own personal countermeasures for a certain length of time.<br>• Alternative countermeasures will be made available should the suggested countermeasures not work well enough for you.	• You will assess the usefulness of the CPoSNS method and whether it helps you to combat procrastination.	• You will be more able to pinpoint the key features of SNS that trigger procrastination and the best countermeasures for you to use.

Guidance for using the D-Crastinate method

1 FIRST STAGE (EDUCATION):

The first stage is to read and familiarise yourself with the key ideas and habits that may lead people to procrastinating more on social networking sites.

2 SECOND STAGE (SELF-DIAGNOSIS):

In this stage you are expected to figure out what kind of procrastinator you are by selecting the types of procrastinating that relate to you specifically. After that you can determine for yourself what you think the main features of social networking sites are that lead you to procrastinate more. However, if you feel like you procrastinate due to other things that are not currently listed you may write them down before moving on to the third stage.

3 THIRD STAGE (PLANNING AND PREPARATION):

This stage has two phases which include tasks engagement tools and procrastination countermeasures. Firstly, if you believe you may procrastinate due to a lack of motivation to complete tasks, please consider using tasks engagement tools. Secondly, you have been given a list of customised countermeasures that can help you to gain more control over how much you procrastinate on a day-to-day basis. They have been customised specifically for you based on the features that lead you to procrastinate.

4 FOURTH STAGE (ACTION):

In this stage you are required to apply the suggested task's engagement tools and the countermeasures for one week.

5 FIFTH STAGE (SELF-ASSESSMENT):

After the action stage is completed, you will be expected to decide how useful you found the previous stages. Move on to the next stage if you do not find the previous stages useful in helping you gain more control over your procrastination.

6 SIXTH STAGE (ERROR IDENTIFICATION):

In this stage you will be expected to answer the provided questions to help you analyse what went wrong in the previous stages. Once you have identified your own personal challenges you can then return to the second stage to apply the method process again.

1 First stage (Education)

In this stage you are expected to get more knowledge about how procrastination usually occurs on social networking sites. This includes general guidance and methods that can help users gain more control over their procrastination.

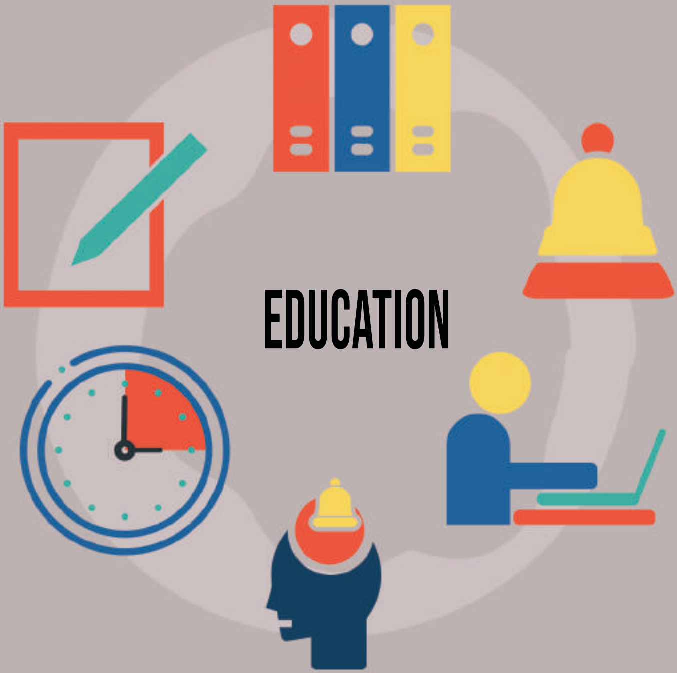

How does procrastination on social networking sites happen?

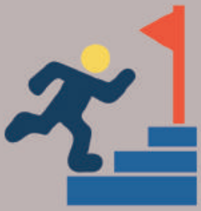

POOR EXPECTATION MANAGEMENT

"My friends often expect me to reply almost instantly when they send me a message, no matter what time."

Many people end up spending large amounts of time procrastinating on social media. Sometimes this can be due to social pressure, usually from friends, who might be very active on social media platforms. In order to maintain a positive self-image some people might try to avoid disappointing their friends by replying to messages and notifications immediately.

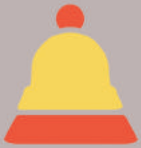

IMPULSIVENESS AND LACK OF SELF-CONTROL

"I lose my control when I see a notification."

People who have a low amount of self-control might react impulsively to their social media notifications. When they see a new notification they might want to respond to it straight away and interact with others. But this might cause them to prioritise social media over tasks they have to do and responsibilities they have to carry out, which will affect how well they work and how they manage themselves in a work environment.

POOR TIME MANAGEMENT

"I have low skills when it comes to managing my time wisely."

People who have low skills in time-management are more likely to spend a lot of time procrastinating. They might tend to forget themselves and get easily distracted when accessing social media instead of prioritising more important tasks, which could lead to them wasting valuable time. These people need to practice managing their time more effectively so they have a healthy balance between carrying tasks / doing homework and spending time on social media (in their free time).

HABITUAL CHECKING

"I check my phone excessively without meaning to do so."

Do you find yourself accessing social media without even intending to do so? The impulse to frequently check social media might lead some people to developing a habit to check their notifications without meaning to, leading to them spending more time than necessary on social media. Habits like this are usually difficult to control as it has become normalised in our society to be constantly using our phones and other devices.

SOCIAL NORMS

"Everyone else is also on their smartphones and end up procrastinating like me."

Some people underestimate their levels of procrastination and might think that their habits are acceptable and normal when they see that others procrastinate also. For example; 'I knew I was going to be late for the lunch event, but everyone was taking a selfie at the venue so I thought it would be okay for me to do the same.'

Solutions for procrastination

To help control your level of procrastination better, you could work to improve your skills in these different areas as follows:

DIGITAL RESILIENCE

If you want to better control your level of procrastination there are tools and information available that can help you. You can find out how procrastination happens, what the negative results of procrastination are, and how to prevent those negative consequences.

Digital resilience means the following:

- You do not get overly affected emotionally by what you see online.
- You understand that a large percentage of what you see online is not necessarily a reflection of the real world.
- You understand how information on social media is filtered to cater to your interests.

ACCEPTANCE OF NON-PERFECT

People who seek to be 'perfect' might feel the need to instantly respond to every single message they receive in order to meet other people's expectations and please them as much as possible. But the pressure many people feel to always be available online might prevent individuals from dealing with their own day-to-day tasks. Being able to accept that you can't please everyone could help you to better manage how much time you spend engaging with people online, and therefore decrease how much time you spend procrastinating on social media.

EXPRESS YOURSELF TO OTHERS

You can manage your friend's expectations by telling them when you are free to interact with them on social media, and when you are busy. Doing this could reduce the pressure you feel to be active on social media too often. You could also apply this procrastination countermeasure with your friends so that you can both motivate each other to spend less time on your devices.

GOAL / LIMIT SETTINGS

Applying goal / limit settings to your life could help you avoid receiving distracting notifications too often. This should eliminate a lot of temptation you may feel to frequently check your social media accounts, and in turn should lead to reducing how much time you spend procrastinating on social media. For example; you could select daily updates instead of hourly ones, or simply mute your notifications. This will help you to develop new, healthy habits and encourage you to engage more with your tasks rather than your devices.

POSITIVE THINKING

Maintaining positive self-talk could help you to curb how much time you spend on social media. E.G; if you had sent a message to someone and found that they had read your message but hadn't yet replied, instead of obsessing over it you could choose to think more positively about the situation; they might have been busy at the time and needed more time to reply. Methods like this also help you develop more understanding and empathy towards others, and help you distance yourself from situations that might otherwise cause you anxiety.

RELAPSE PREVENTION

Once you decide to take control back into your own hands and spend less time procrastinating on social media, you must prepare for there to be a relapse. It can help to understand what the signs are. how it happens and what strategies you can implement into your life to help you either avoid it or tackle it. The relapse occurs when you fail to use the suggested techniques to help you control your procrastination levels, and you ultimately return to your previous habits of spending too much time on social media.

THE RELAPSE OCCURS IN THREE STAGES:

- Emotional relapse when you think about how spending time on social media could help you feel better and improve your mood.
- Mental relapse when you struggle to train yourself into spending less time on social media.
- Physical relapse when you return back to procrastinating like you did before.

In order to prevent or help this relapse it might help you to:

- List things that will help motivate you to beat procrastination.
- List the negative results of procrastination that you want to avoid.

2 Second stage (Self-Diagnosis)

TYPES OF PROCRASTINATION

Avoidance	Mood modification	Escapism	Emergence
I often procrastinate to avoid working on unpleasant or difficult tasks.	I often procrastinate to change my mood and to feel better.	I often procrastinate to distance myself from real-life issues.	When I receive a notification I check it automatically and spend time on that, despite having other tasks to perform.

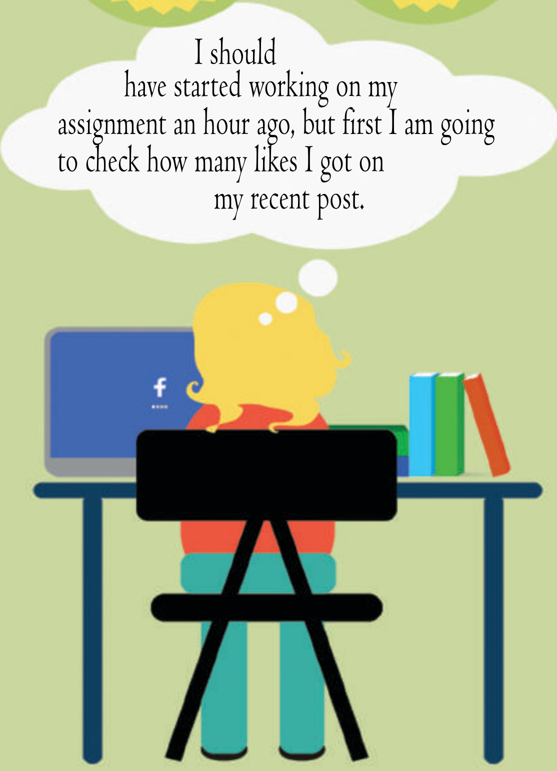

NOTE: People might also procrastinate due to peer pressure, to increase their popularity on social networking sites, and to help them stay up-to-date with what's happening on social media. Describe your own reasons for procrastination if you feel the suggested types do not apply to you:

2 Second stage (Self-Diagnosis)

THE FEATURES OF SOCIAL NETWORKING SITES THAT MAY INCREASE PROCRASTINATION

I often get distracted by notifications which lead me to accessing my social media and then delaying working on my tasks.

Notification

When I send a message to someone, I keep checking whether or not they are active online.

Surveillance of presence

I respond to my contacts on social media almost instantly in order to build a positive self-image and maintain a good profile.

Identity

I find it hard to pull myself away from online conversations in order to complete my tasks.

Interaction

While on social media I often see suggested content that is relevant to me, and I end up spending more time than I intended to spend on those sites.

Immersive Design

NOTE: If you feel the above suggested features of SNS do not lead you to procrastinate, list below some other features you can think of that do apply to you:

3 Third Stage (Planning and preparation)

TASK ENGAGEMENT TOOL

Please identify a reward system for yourself that will help you towards achieving your goals. For example,; if you successfully managed to avoid using social media in class for one full week you could reward yourself by doing something you enjoy such as having lunch outside or going to the cinema.

Instead of trying to accomplish one huge task, break it up into multiple goals that you can easily achieve, and arrange realistic deadlines for each milestone.

Make your contacts aware that you can't be available online all the time due to commitments you have and tasks you have to accomplish. This could lessen the pressure you feel to interact constantly on social media.

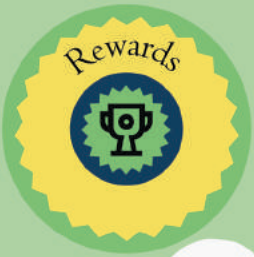

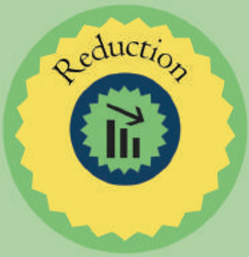

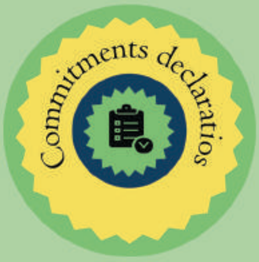

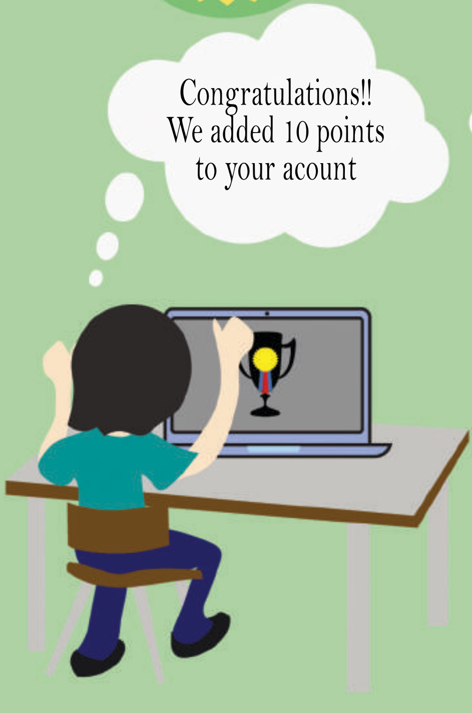

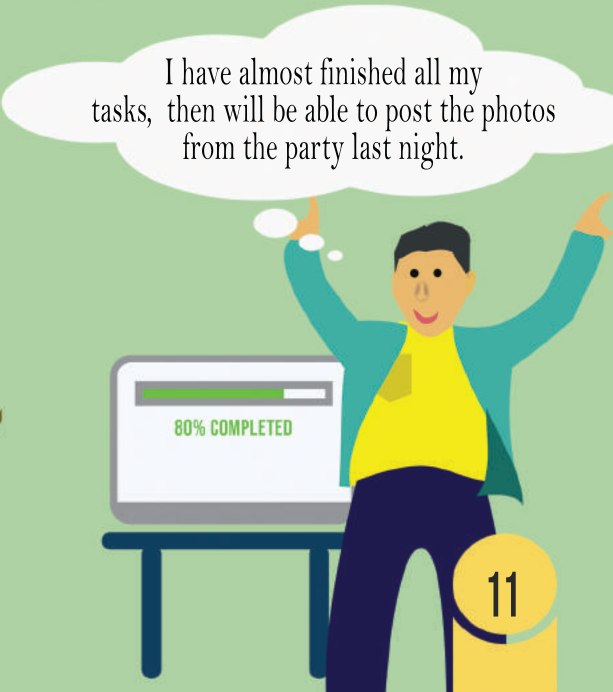

Third Stage (Planning and preparation)

TECHNICAL COUNTERMEASURES AND SOCIO-TECHNICAL COUNTERMEASURES PHASE

When you feel you are likely to procrastinate due to checking your notifications and engaging with their content, which of these software techniques would help you to control your procrastination? (You can choose more than one.)

Set up auto-reply so that your contacts are automatically informed about your availability when they message you.

Auto-reply — SOCIO-TECHNICAL

Set a status to show your contacts when you are not available so they do not expect you to interact immediately.

Showing availability — SOCIO-TECHNICAL

Seek advice and suggestions if are not sure how to do various technical things such as muting notifications or setting a timer. For example, in iOS you could use Siri to get more suggestion.

Suggestions — TECHNICAL

When you spend more time on social media than you initially intended, which of these software techniques would best help you to combat that?

Set a reasonable time limit that you wish to not exceed on social media. For example, using screen apps limit or down time in iOS. To do so, go to the setting in iOS >> screen times >> app limits or down time.

Time restriction — TECHNICAL

Decide on an amount of time that you wish to spend on social media. For example, if you wish to spend one hour per day on social media you could set up a timer for one hour so that you would receive a reminder when the timer is up

Usage reminder — TECHNICAL

Please, utilise your usage feedback that you have spent on social media during your working hours. For example in iOS, go to setting>>screen time>> usage feedback.

Usage feedback — TECHNICAL

Third Stage (Planning and preparation)

TECHNICAL COUNTERMEASURES AND SOCIO-TECHNICAL COUNTERMEASURES PHASE

When you send a message to someone and you find yourself constantly checking to see if they are active online, which of these software techniques would help you to combat that procrastination?

Set up auto-reply to respond automatically to your contacts in order to let them know about your availability time. For example, set up an auto-reply for your email account.

Auto-reply
SOCIO-TECHNICAL

Prioritise your tasks based their importance to you. For example, list tasks that you need to carry out this week, ranking them in order of urgency, and then prioritising them over other things.

Tasks priority
TECHNICAL

When you procrastinate by replying to your contacts as soon as they message you, mainly to build a positive self-image and maintain a good profile, which of these software techniques would help you to tackle that procrastination?

Please, utilise your usage feedback that you have spent on social media during your working hours. For example in iOS, go to setting>>screen time>> usage feedback.

Usage feedback
TECHNICAL

Decide on a time limit that you wish to not exceed on social media. For example, using screen apps limit or down time in iOS. To do so, go to settings in iOS>> screen times>>app limits or down time.

Time restriction
TECHNICAL

Set up auto-reply to respond automatically to your contacts in order to let them know about your availability time. For example, set up an auto-reply for your email account so you don't feel the need to reply to everyone immediately

Auto-reply
SOCIO-TECHNICAL

Set personal goals that you wish to achieve. For example, if you wished to study for one hour per day, you could set up a timer for one hour each time you study so as to organise your time more effectively.

Goal setting
TECHNICAL

Third Stage (Planning and preparation)

TECHNICAL COUNTERMEASURES AND SOCIO-TECHNICAL COUNTERMEASURES PHASE

When you prioritise chatting to your friends on social media instead of focusing on your tasks, which of these software techniques would help you to combat that problem?

Set up the time you wish to spend messaging your contacts on social media and let them know about the suggested time. For example, if you wish to chat for ten minutes you could set up a timer for ten minutes when the conversation starts with each of your contacts.

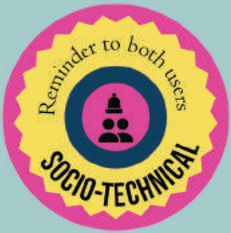

Please set a status to show your contacts when you are not available so they do not expect you to interact with them at certain times when you are busy.

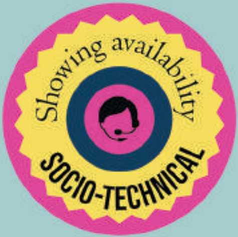

Decide on the amount of time that you wish to spend engaging with other online. For example, if you wished to chat for ten minutes you could set up a timer for ten minutes when the conversation is starts.

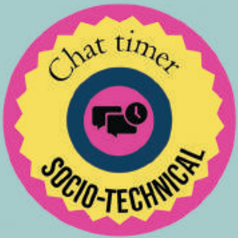

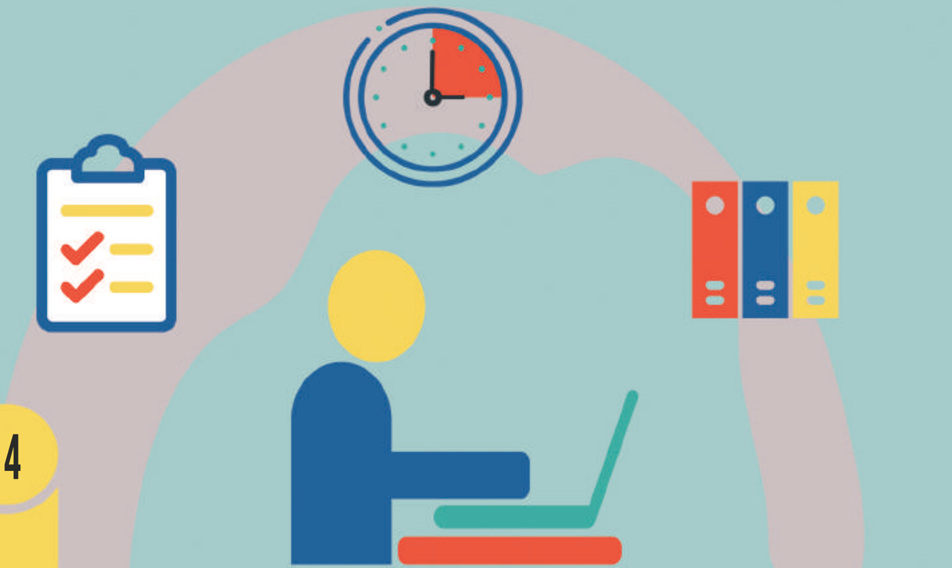

Please apply the selected countermeasures for one week.

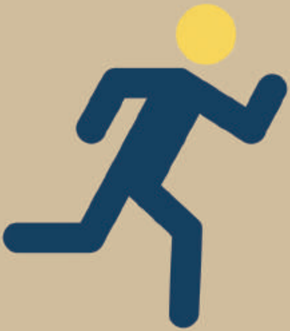

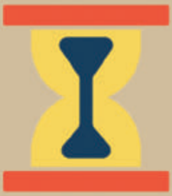

Alternative countermeasures will be made available should the suggested countermeasures not work well enough for you.

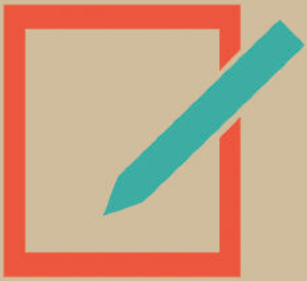

5 Fifth stage (Self-assessment)

Answer the question:

Do the suggested countermeasures help you to gain more control over your procrastination?

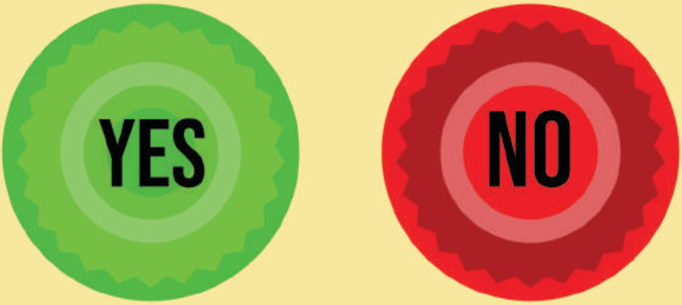

6 Sixth stage (Error identification)

On a separate sheet please answer the following questions to the best of your ability:

Where did you procrastinate? (In which application)

When did you procrastinate? (What time)

What did you procrastinate? (Tasks that you missed)

Why did you procrastinate? (Your reasons for procrastinating)

How did you procrastinate? (Other activities you did while procrastinating)

Who did you procrastinate with? (Other people who were involved and who were perhaps affected by your procrastination)

NOTE: Once you have answered the previous questions you will be able to select more specific countermeasures to help control your procrastination. Also, if you find you end up procrastinating because of other people (peer pressure), the socio-technical countermeasures would perhaps be more helpful than the technical countermeasure to aid you in controlling how much you procrastinate. However, if you feel you procrastinate because of low self-control skills or poor time management you might instead consider the technical countermeasures.

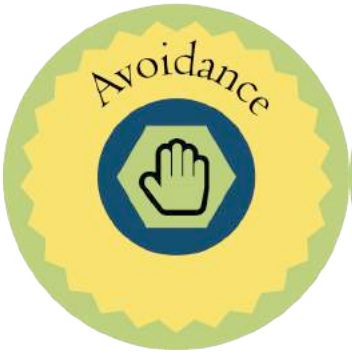
Avoidance
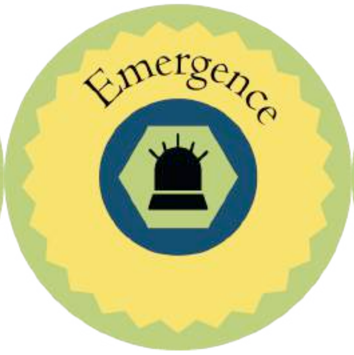
Emergence
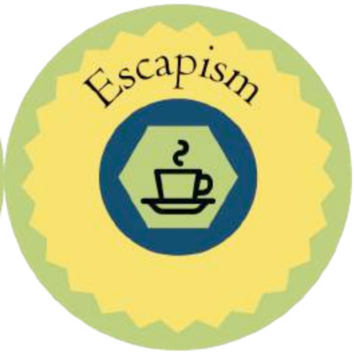
Escapism
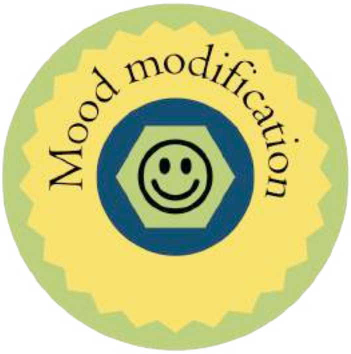
Mood modification
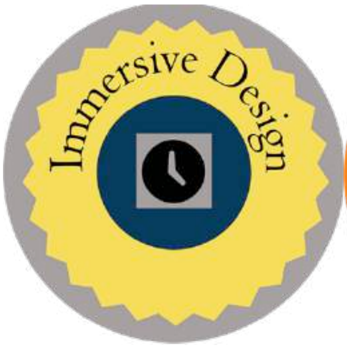
Immersive Design
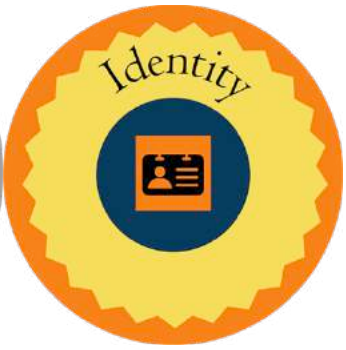
Identity
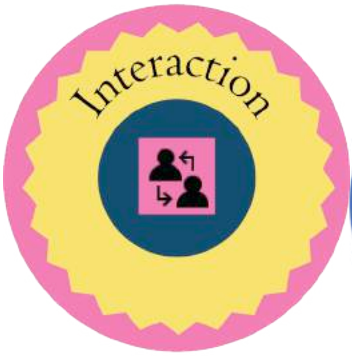
Interaction
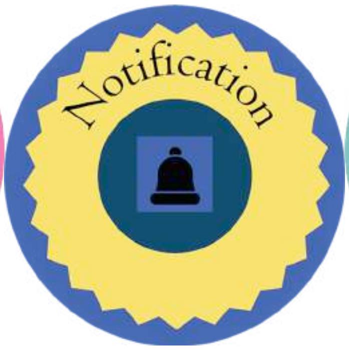
Notification
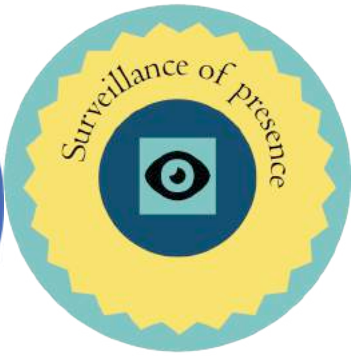
Surveillance of presence
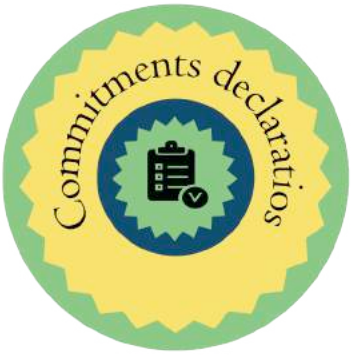
Commitments declaratios
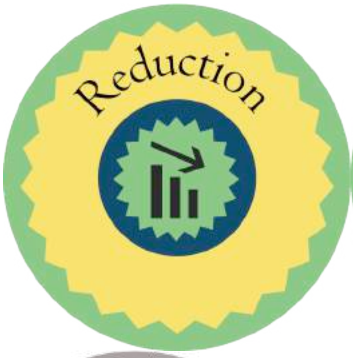
Reduction
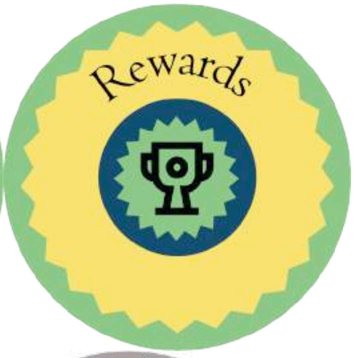
Rewards
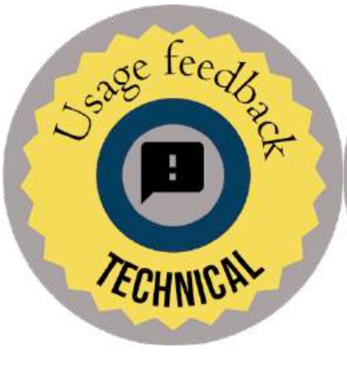
Usage feedback
TECHNICAL
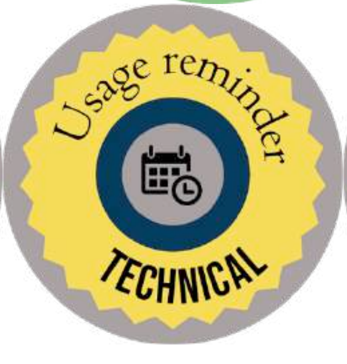
Usage reminder
TECHNICAL
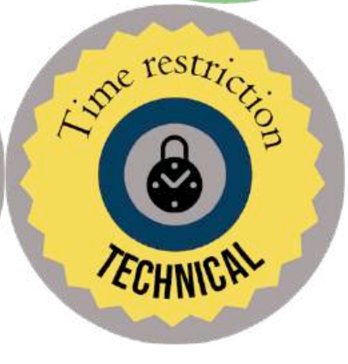
Time restriction
TECHNICAL

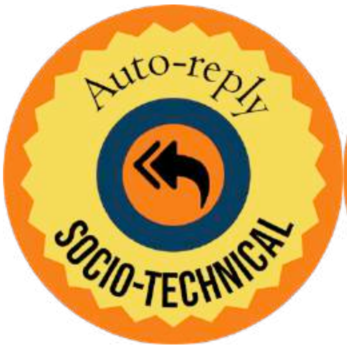
Auto-reply
SOCIO-TECHNICAL

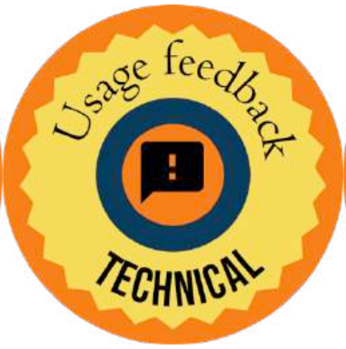
Usage feedback
TECHNICAL

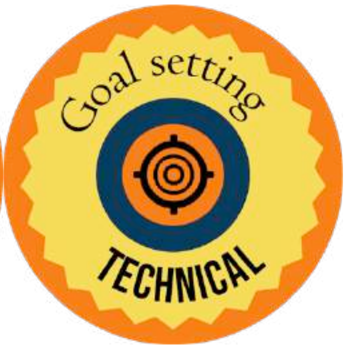
Goal setting
TECHNICAL

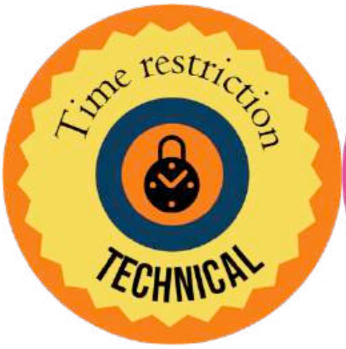
Time restriction
TECHNICAL

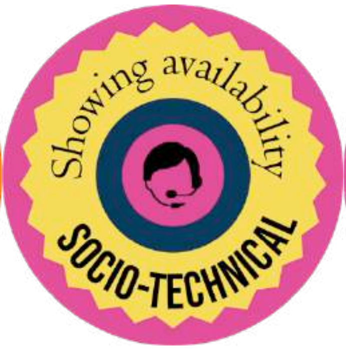
Showing availability
SOCIO-TECHNICAL

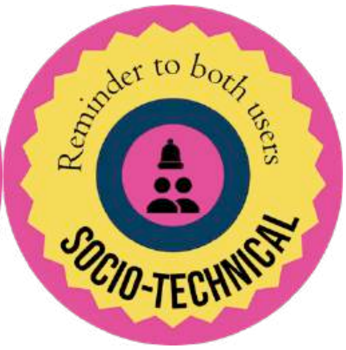
Reminder to both users
SOCIO-TECHNICAL

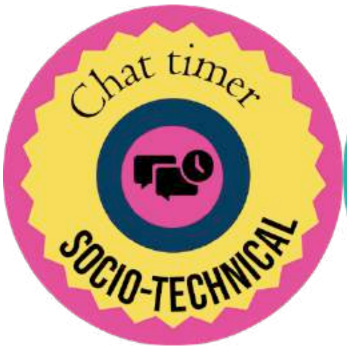
Chat timer
SOCIO-TECHNICAL

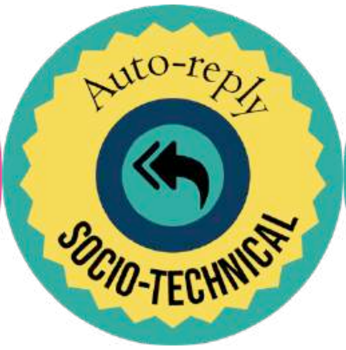
Auto-reply
SOCIO-TECHNICAL

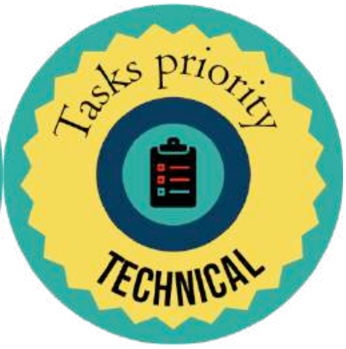
Tasks priority
TECHNICAL

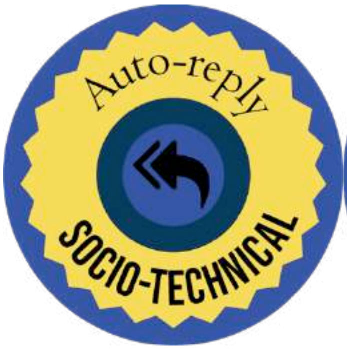
Auto-reply
SOCIO-TECHNICAL

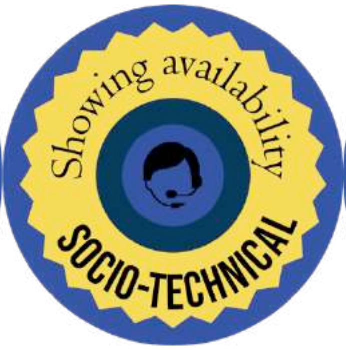
Showing availability
SOCIO-TECHNICAL

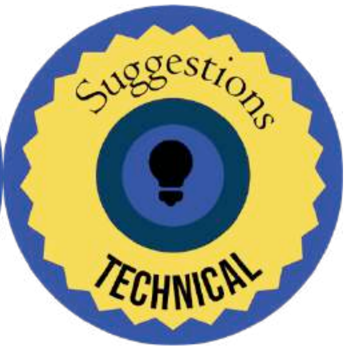
Suggestions
TECHNICAL

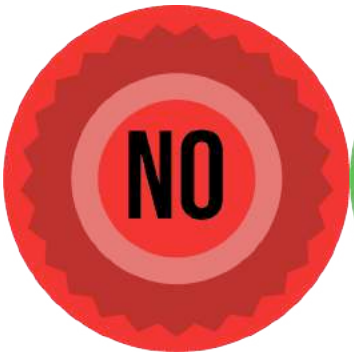
NO

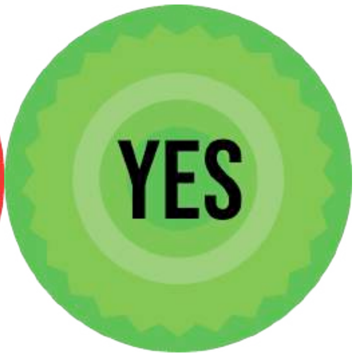
YES

SELF - MONITORING SHEET

My procrastination type(s) are :

Social media feature(s) that lead me to procrastination are:

To increase my task's engagement, I will use the following tools:

To gain better control over my procrastination I will use the following countermeasure(s):

Do the suggested countermeasures help you to gain more control over your procrastination? :
